# The involvement of microglia and the CXCL16-CXCR6 axis in the recruitment of CD8^+^ T cells to an amyloidogenic mouse brain

**DOI:** 10.1038/s41598-025-22137-5

**Published:** 2025-10-31

**Authors:** Marco Zattoni, Sabine Bernegger, Sofia Weinbender, Barbara Altendorfer, Heike Mrowetz, Ariane Benedetti, Rodolphe Poupardin, Michael Stefan Unger, Ludwig Aigner

**Affiliations:** 1 Institute of Molecular Regenerative Medicine , Paracelsus Medical University , Salzburg, Austria; 2 Institute of Experimental Neuroregeneration , Paracelsus Medical University , Salzburg, Austria; 3 Institute of Experimental and Clinical Cell Therapy , Paracelsus Medical University , Salzburg, Austria; 4 Austrian Cluster for Tissue Regeneration, Vienna, Austria

**Keywords:** Cxcr6, Cxcl16, CD8^+^ T cells, Microglia, Alzheimer’s disease, Immunology, Molecular biology, Neuroscience

## Abstract

Alzheimer’s disease (AD) progression has been associated with the presence of brain-resident CD8^+^ T cells, and recent studies suggest a potential role of the CXCL16-CXCR6 axis in their recruitment to the brain. Here, we examined publicly available single-cell RNA sequencing datasets revealing that in the mouse brain, the receptor *Cxcr6* is mainly expressed by CD8^+^ T cells, while the expression of its ligand *Cxcl16* is predominantly observed in microglial cells. We found higher levels of *Cxcl16* and *Cxcr6* expression in APP/PS1 compared to wild-type mice. Furthermore, *in vitro* experiments using immortalized and primary murine cells suggested that Cxcl16 expression is driven by Aβ pathology. In contrast to our expectations, no changes in the number of Cxcr6^+^CD8^+^ cells was evident in the brains of microglia-depleted APP/PS1 mice, treated with the CSF1R antagonist PLX5622. This was related to an increased compensatory Cxcl16 expression by depletion-resistant microglia or by other brain-resident myeloid cells. Although we demonstrated a strong association between microglial Cxcl16 and AD pathology, PLX5622-sensitive microglia are dispensable in the recruitment of Cxcr6^+^CD8^+^ T cells to the brain of APP/PS1 mice. Future *in vivo* analysis will help to dissect the mechanism of CD8^+^ T cell recruitment to the brain.

**Supplementary Information:**

The online version contains supplementary material available at 10.1038/s41598-025-22137-5.

## Introduction

The classic neuropathological hallmarks of Alzheimer’s disease (AD) include the deposition of brain amyloid-β (Aβ) aggregates in the form of senile plaques and intracellular accumulation of hyperphosphorylated tau protein, which constitutes the characteristic neurofibrillary tangles^1,2^. However, neuroinflammation has been increasingly recognized to play crucial roles in AD progression^3–5^.

Seminal studies have demonstrated that, in addition to astrogliosis and microglial activation, other inflammatory processes provoke an adaptive immune response in AD. This includes the infiltration of CD8^+^ T cells into the disease-affected brain parenchyma^6–11^. Immunohistochemical (IHC) analysis of post-mortem brain specimens from AD-affected patients and AD-mouse models revealed a close contact of CD8^+^ T cells with microglia and neurons^6,12^. However, the molecular and cellular mechanism involved in the recruitment of CD8^+^ T cells to the brain parenchyma of AD individuals has not been clarified yet.

Recent data suggest the involvement of the CXCL16-CXCR6 axis in CD8^+^ T cell recruitment and accumulation in the diseased brain. The human transmembrane multi-domain chemokine (C-X-C motif) Ligand 16 (CXCL16), typically acts in its soluble form as a chemoattractant for Chemokine (C-X-C motif) Receptor 6 (CXCR6)-expressing lymphocytes^13–15^. In its membrane bound form, CXCL16 serves as a cell-cell-adhesion molecule binding to CXCR6 molecules expressed on the surface of attracted cells^15,16^. CXCR6, the only known receptor for CXCL16, is the classical homing receptor expressed on human tissue resident memory T (T_RM_) cells^17^.

Single-cell RNA sequencing (scRNA-seq) analysis of mice infected with central nervous system (CNS)-trophic virus, and cerebrospinal fluid (CSF) of cognitively impaired patients, indicated a potential contribution of the CXCL16-CXCR6 axis to the accumulation of CD8^+^ T cells in the brain^18,19^. Moreover, in a recent transcriptomic analysis on murine brain-isolated CD3^+^CD8^+^ T cells, we demonstrated higher Cxcr6 (the murine orthologue of CXCR6) expression levels compared to their blood counterparts. In particular, counting of hippocampal Cxcr6^+^CD8^+^ cells revealed higher numbers of this T cell subtype in APP/PS1 mice, a widely used animal model of AD^20^. Thus, CD8^+^ T cells expressing CXCR6 might infiltrate the brain via the CXCL16-CXCR6 axis as a potential molecular mechanism. Consequently, gaining insight into the cellular origin of CXCL16 within the brain would enhance our understanding of the mechanisms underlying the infiltration of parenchymal CD8^+^ T cells.

Here, we aim to assess the interplay between Cxcr6^+^CD8^+^ cells and Cxcl16 (the murine orthologue of CXCL16)-producing cells in the APP/PS1 brain. To this end, we analyzed previously published scRNA-seq murine datasets. We then performed quantitative PCR (qPCR), Western Blot (WB) and Enzyme-Linked Immunosorbent Assay (ELISA) on Aβ-treated immortalized and primary microglia and macrophage cell cultures, to assess Cxcl16 expression levels in *in vitro* AD-like conditions. Subsequently, we analyzed the brains of mice treated with PLX5622, a microglia-depleting agent inhibiting colony-stimulating factor 1 receptor (CSF1R/Csf1r), to investigate the role of microglia-produced Cxcl16 in the recruitment of Cxcr6^+^CD8^+^ cells *in vivo*. Finally, by taking advantage of other scRNA-seq datasets, we hypothesisze the existence of a compensatory mechanism that supports Cxcl16 expression following the pharmacological and genetic inhibition of Csf1r.

## Results

### The cellular sources of *Cxcr6* and *Cxcl16* in brain immune cells

**Fig. 1:**
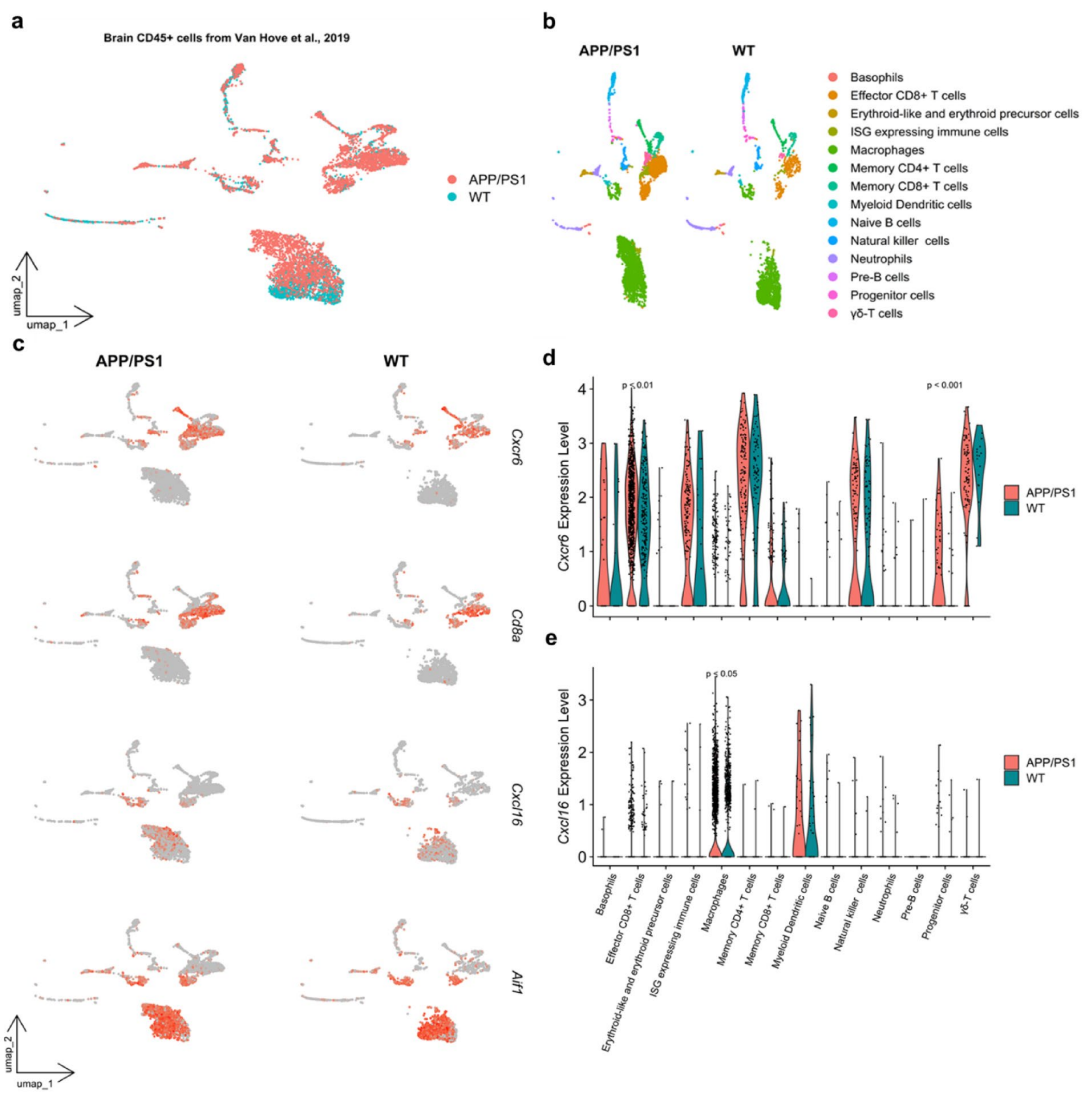
***Cxcr6 * and** ***Cxcl16*** **expression in scRNA-seq from brain-derived CD45**^+^ **cells.** **a**) UMAP showing the clustering of CD45^+^ brain-derived cells from 16 months APP/PS1 and WT animals in Van Hove *et al.,* 2019. **b**) UMAP showing the color-coded clustering of the cells, subdivided in APP/PS1 (left plot) and WT (right plot) 16-month-old animals. **c**) Feature plots of CD45^+^ brain-derived cells from 16-month-old APP/PS1 and WT animals showing the expression of selected genes (*Cxcr6*, *Cd8a*, *Cxcl16* and *Aif1*), where red color indicates cells showing highest levels of expression. **d**, **e**) Violin plots showing the expression of *Cxcr6* (**d**) and *Cxcl16* (**e**), between 16-month-old APP/PS1 and WT animals, in the different cell clusters. The dots indicate the cells, expressing the transcripts, within each cluster. Statistical analysis was performed with Wilcoxon test. *p* < 0.05 was considered statistically significant and displayed in the plot.

We re-clustered scRNA-seq data of CD45^+^cells, sorted from brain tissue of APP/PS1 and WT animals^21^, using unsupervised graph-based clustering by Seurat (Fig. 1a). Cell type annotation was automatically performed using comprehensive immune system cell markers database^22^, (Fig. 1b). Cell clusters percentage revealed genotype-related differences, especially for the “Effector CD8^+^ T cells” cluster, which showed increased proportion in the APP/PS1 brain (Supplementary Figure S1a). Interestingly, re-clustering and automated cell-based annotation of CD45^+^ cells from 9- and 16-month-old APP/PS1 mice revealed a higher proportion of “Effector CD8^+^ T cells” in a later stage of the disease, confirming our recent data^20^ (Supplementary Figure S1b-d).

UMAP plots showed that in 16-month-old animals *Cxcr6* transcript is mainly co-expressed together with the cytotoxic T cell marker *Cd8a*, while *Cxcl16* expression is characteristic of brain macrophages identified by the expression of *Aif1*, which encodes for the microglial marker Iba1 (Fig. 1c). Violin plots further highlighted an increased presence of *Cxcr6-*expressing cells among the “Effector CD8^+^ T cells” (Wilcoxon test, *p* < 0.01) and the “Progenitor cells” (abbreviation for “Hematopoietic progenitor cells”, Wilcoxon test, *p* < 0.001) clusters in APP/PS1 mice compared to WT controls (Fig. 1d). Similarly, *Cxcl16* expression is statistically significant expressed at higher levels in “Macrophages” cells from APP/PS1 compared to WT mice (Wilcoxon test, *p* < 0.05) (Fig. 1e).

Genotype specific differences are denoted also by the increased cell proportion and total amount of cells expressing *Cxcr6* and *Cxcl16* in APP/PS1 compared to WT brain (Supplementary Table S1−2).

### Aβ pathology influences Cxcl16 expression *in vitro* and *in vivo*

**Fig. 2:**
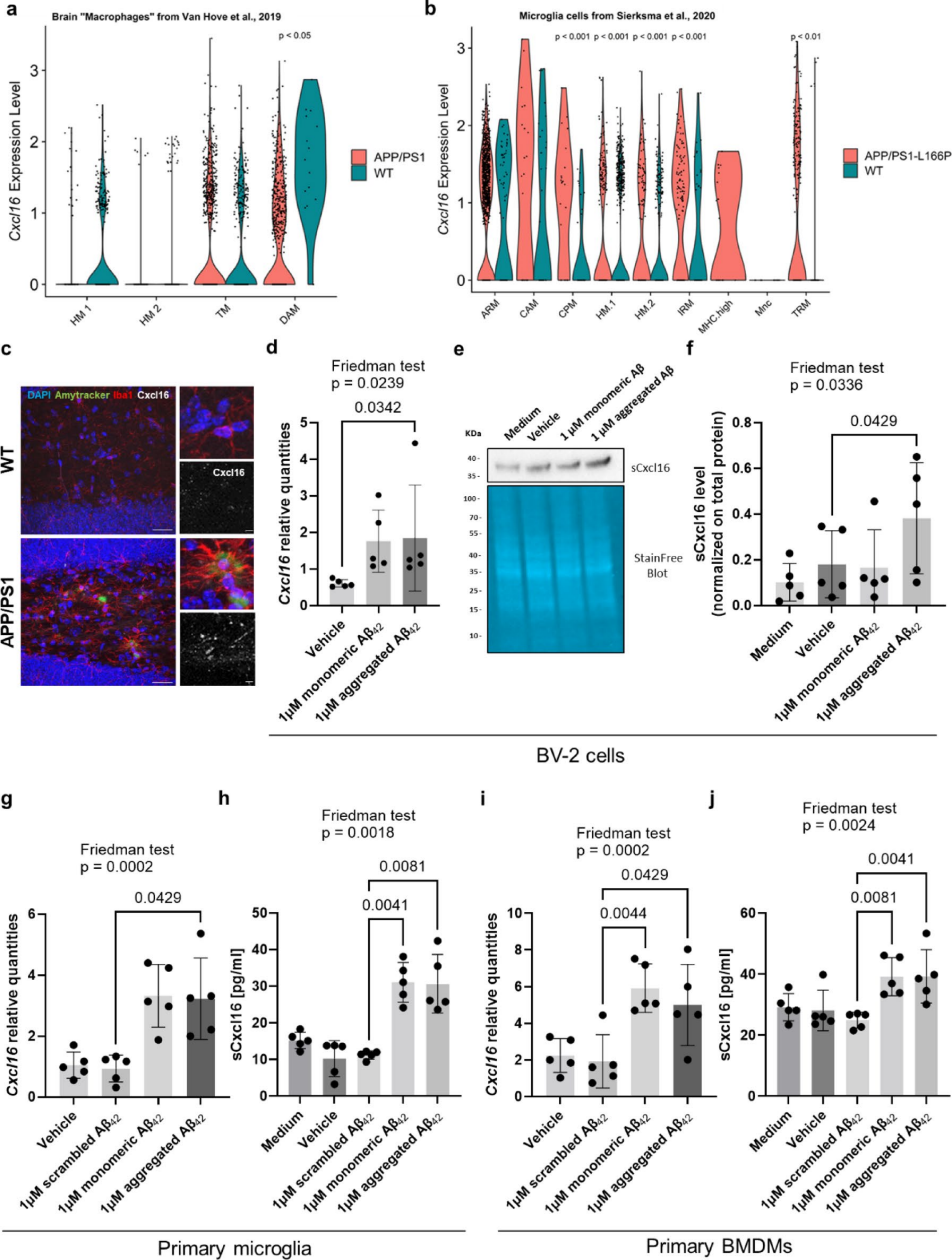
**Aβ pathology effect on Cxcl16 microglial expression** ***in vitro*** **and** ***in vivo. *a**, **b**) Violin plots showing the expression of *Cxcl16* between 16-month-old APP/PS1 and WT animals, in Van Hove *et al.,* 2019 “Macrophage” cell cluster (**a**, “Homeostatic Microglia 1” (HM 1), “Homeostatic Microglia 2” (HM 2), “Transitioning Microglia” (TM), “Disease-associated Microglia” (DAM)), and between 11-month-old APP/PS1-L166P and WT animals, in Sierksma *et al.,* 2020 dataset (**b**, “Activated Response Microglia” (ARM), “CNS-Associated Macrophages” (CAM), “Cycling and Proliferating Microglia” (CPM), “Homeostatic Microglia Cluster 1” (HM.1), “Homeostatic Microglia cluster 2” (HM.2), “Interferon-Response Microglia” (IRM), “High Major Histocompatibility Complex-expressing Microglia” (MHC.high), “Monocytes” (Mnc), “Transitioning Microglia” (TRM)). The dots indicate the cells, expressing the transcripts, within each cluster. Statistical analysis was performed with Wilcoxon test. p < 0.05 was considered statistically significant and displayed in the plot. **c**) Representative image of granule cell and polymorph layers of the dentate gyrus in hippocampal region of WT and APP/PS1 animals. C*xcl16* signal is in white, Iba1 (red) was used to stain microglia cells, DAPI (blue) stains the nucleus, and Amytracker 520 (green) is used to visualize Aβ plaques. Scale bar full picture: 50 µm, scale bar zoom: 10 µm. **d**, **g**, **i**) qPCR analysis for *Cxcl16* mRNA expression level (“relative quantities” normalized to medium-treated cells) in BV-2 cells (**d**) treated with vehicle, 1 μM monomeric and 1 μM aggregated Aβ_42_ and in primary microglia (**g**) and bone marrow-derived macrophages (BMDMs, i) treated also with 1 μM scrambled Aβ_42_. **e**) Representative WB image secreted Cxcl16 (sCxcl16) in supernatants from BV-2 cells treated with medium, vehicle, 1 μM monomeric and aggregated Aβ_42_ for 24 h. Stain-free Blot signals was used as protein loading control. Molecular weights in kilo Dalton (kDa) are indicated on the right. **f**) Densitometric analysis of Stain-free-signal-normalized sCxcl16 levels in medium alone, vehicle, 1 μM monomeric and 1 μM aggregated Aβ_42_. **h**, **j**) ELISA-based quantification of sCxcl16 in pg/mL in the cell supernatant of primary microglia (**h**) and BMDMs (**j**) treated with medium, vehicle, 1 μM scrambled Aβ_42_, 1 μM monomeric and 1 μM aggregated Aβ_42_. Statistical analysis in **d**, **f**, **g**-**j** (n = 5 repeated experiments) was performed with the Friedman test with Dunn's multiple comparison test (exact *p* value was calculated, and *p* values < 0.05 were considered statistical significant and displayed in the plot).

To dissect the origin of *Cxcl16* transcripts, we sub-clustered the “Macrophages” cell population, using previously established microglia/brain macrophage markers listed in the heat map (Supplementary Figure S2a-c). Violin plot revealed increased *Cxcl16* expression levels in APP/PS1 “Disease-Associated Microglia” cell cluster (DAM, Wilcoxon test, *p* < 0.05) compared to WT (Fig. 2a), suggesting Cxcl16 as a signature marker of AD-related microglia states in APP/PS1 brain tissue.

To explore the microglia-specific expression of *Cxcl16* in the context of AD, we took advantage of a scRNA-seq dataset of CD11b^+^/CD45^+^ microglia isolated from the brain of the APP/PS1-L166P mouse model of AD, published by Sierksma *et al*., 2020^23^. As expected, UMAP analysis revealed a marked genotype difference between APP/PS1-L166P and WT animals (Supplementary Figure S2d, e). *Cxcl16* was expressed higher in all microglial subtypes of APP/PS1-L166P brain, with a statistically significant difference among “Cycling and proliferating microglia” (CPM, Wilcoxon test, *p* < 0.01), “Homeostatic microglia cluster 1” (HM.1, Wilcoxon test, *p* < 0.01), “Homeostatic microglia cluster 2” (HM.2, Wilcoxon test, *p* < 0.01), “Interferon-response microglia” (IRM, Wilcoxon test, *p* < 0.0001) and “Transitioning microglia” (TM, Wilcoxon test, *p* < 0.05) clusters (Fig. 2b), suggesting that *Cxcl16* overexpression characterized both physiological and disease-associated microglial states in the APP/PS1-L166P model. The AD pathology-dependent microglial *Cxcl16* expression is further supported by the IHC analysis of APP/PS1 dentate gyrus, which revealed the presence of Cxcl16 signal (white) in the proximity of Iba1^+^ microglia cells (red) around Aβ plaques (green) (Fig. 2c). To further understand whether *Cxcl16* expression in microglial cells is affected by AD pathology, we exposed immortalized murine microglial (BV-2) cells to monomeric and aggregated Aβ_42_ peptides (Supplementary Figure S3a, the uncropped WB image is available in Supplementary Figure S4a). First, we confirmed a pro-inflammatory response to Aβ exposure of the BV-2 microglia, as demonstrated in the 2-fold increase of *Tnf* expression by qPCR (Friedman test, *p* = 0.0239, Dunn’s multiple comparisons test between vehicle- and aggregated Aβ_42_-treated cells, *p* = 0.0228, Supplementary Figure S3b). 24 h treatment with 1 µM of aggregated Aβ_42_ led to statistical significant increased expression of *Cxcl16* transcript compared to vehicle-treated BV-2 cells (Friedman test, *p* = 0.0239, Dunn’s multiple comparisons test, *p* = 0.0342, Fig. 2d). To observe whether the change in mRNA expression is also reflected at the protein level, we performed WB analysis on monomeric and aggregated Aβ_42_-exposed BV-2 cells. Non-specific bands appeared in the WB analysis of the intracellular form of Cxcl16 (Supplementary Figure S4b). Subsequent experiments were therefore performed on the cell culture supernatant to analyse the secreted form of Cxcl16 (sCxcl16) (Fig. 2e, original WB images are available in Supplementary Figure S4b). Densitometric analysis revealed a statistically significant increase in sCxcl16 after the treatment with 1 µM aggregated Aβ_42_ (Friedman test, *p* = 0.0336, Dunn’s multiple comparisons test, *p* = 0.0429, Fig. 2f), suggesting an Aβ_42_ structure-dependent release of sCxcl16 from treated BV-2 cells.

To strengthen our hypothesis, we verified the expression level of Cxcl16 in a primary murine microglia cell culture system, since this more closely resembles the environment of the brain. Purity of mouse primary microglia cultures was assessed via Cd11b expression (Supplementary Figure S5a, b). In these experiments, we included a scrambled Aβ_42_ sequence to provide a more reliable control to evaluate the effect of Aβ_42_ peptide. Exposure to both monomeric and aggregated Aβ_42_, resulted in increased *Tnf* expression compared to scrambled Aβ_42_, with a statistical significant effect between scrambled and monomeric Aβ_42_ sequences (Friedman test, *p* < 0.0001, Dunn’s multiple comparisons test, *p* = 0.0212, Supplementary Figure S3c). Similarly, *Cxcl16* transcript levels increased after treatment with monomeric and aggregated Aβ_42_. However, a statistically significant effect was only observed between scrambled and aggregated Aβ_42_ (Friedman test, *p* = 0.0002, Dunn’s multiple comparisons test, *p* = 0.0429, Fig. 2g), supporting the effect observed in BV-2 cells. We then quantified sCxcl16 in the supernatant of primary microglia treated with Aβ_42_ using ELISA, a more robust approach than WB for quantifying chemokines. The results indicated that both monomeric and aggregated forms induced a statistically significant increase in sCxcl16 when compared to scrambled Aβ_42_ sequence (Friedman test, *p* = 0.0018, Dunn’s multiple comparisons test, *p* = 0.0041 and *p* = 0.0081, respectively, Fig. 2h).

Further experiments were conducted using mouse bone marrow-derived macrophages (BMDMs), which express the typical macrophage markers CD11b and F4/80 (Supplementary Figure S5c, d), in order to establish whether the response to monomeric and aggregated Aβ_42_ is confined to CNS resident microglia cells. While these cells did not respond to Aβ_42_ in the same way as primary microglia in terms of *Tnf* expression (Friedman test, *p* = 0.2096, Supplementary Figure S3d), they exhibited significant *Cxcl16* transcript upregulation upon exposure to monomeric and aggregated Aβ_42_ compared to the scrambled sequence (Friedman test, *p* = 0.0002, Dunn’s multiple comparisons test, *p* = 0.0044 and *p* = 0.0429, respectively, Fig. 2i). Furthermore, these gene expression changes were confirmed at the protein level. We observed a statistically significant increase in sCxcl16 in the supernatant of BMDMs exposed to both monomeric and aggregated Aβ_42_ when compared to scrambled control (Friedman test, *p* = 0.0024, Dunn’s multiple comparisons test, *p* = 0.0081 and *p* = 0.0041, respectively, Fig. 2j).

Overall, these *in vitro* experiments support the hypothesis that Cxcl16 expression in CNS-resident and peripheral macrophages depends on AD pathology.

### Expression of *Cxcl16* and *Cxcr6* is elevated in APP/PS1 hippocampus and is not altered upon PLX5622 treatment

**Fig. 3:**
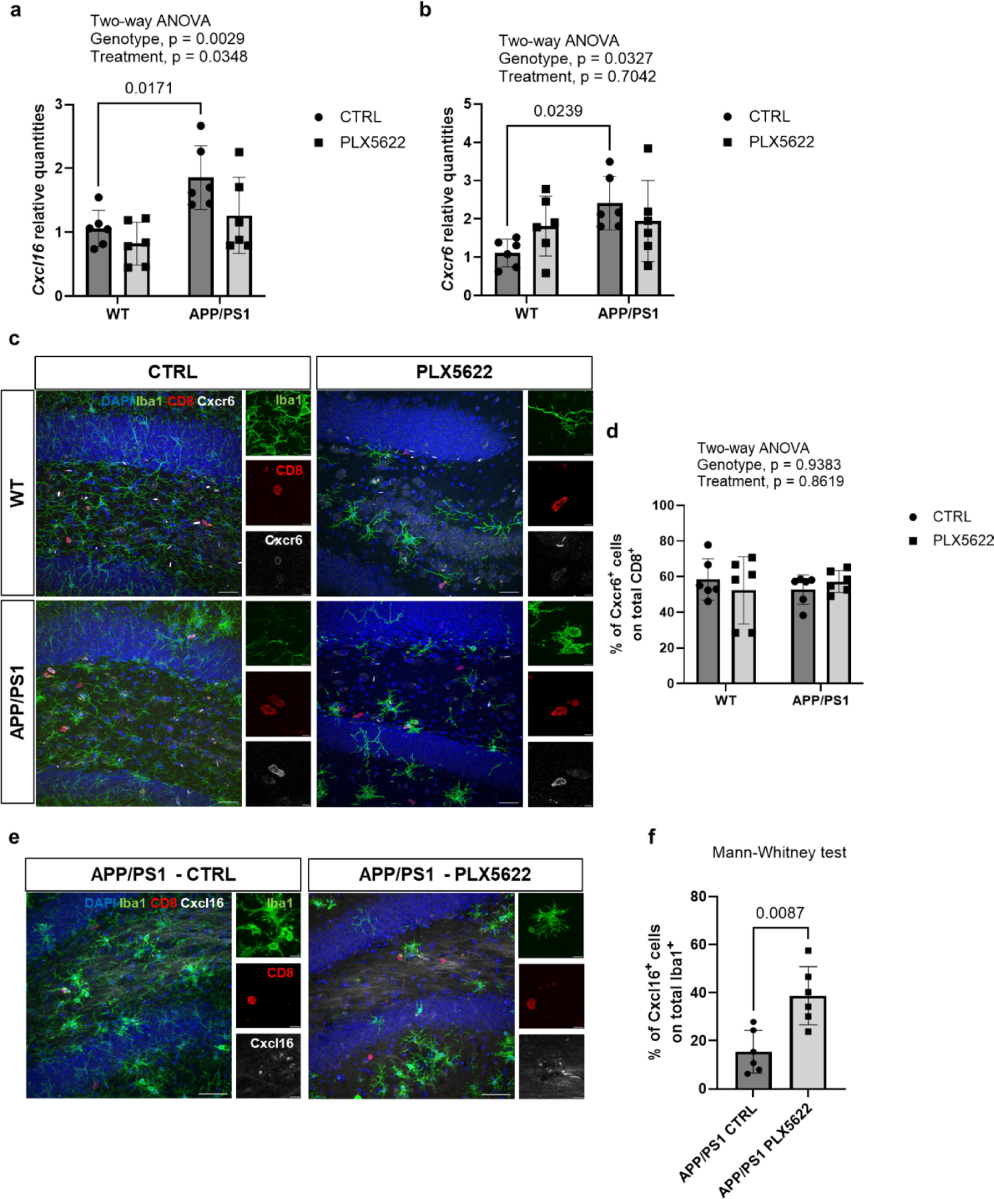
**The role of the Cxcl16-Cxcr6 axis in the hippocampus of amyloidogenic mice after PLX5622-mediated microglia depletion**. **a**, **b**) qPCR analysis for *Cxcl16* (**a**) and *Cxcr6* (**b**) mRNA expression level (“relative quantities”) in 12-month-old control (CTRL) and microglia-depleted (PLX5622) WT and APP/PS1 animals (n = 6 each group). Statistical analysis was performed with the Two-way ANOVA with Šídák's multiple comparisons test (exact *p* values were calculated, and *p* values < 0.05 were considered statistical significant and displayed in the plots). **c**) Representative images of granule cell and polymorph layers of the dentate gyrus in the hippocampal region of PLX5622-treated and control WT and APP/PS1 animals. Cxcr6 signal is in white, CD8^+^ cells are in red, DAPI (blue) stains the nucleus, and Iba1 (green) is used to visualize microglia cells. Scale bar full picture: 50 µm, scale bar zoom: 10 µm. **d**) Quantitative analysis of the percentage of cells expressing Cxcr6 on the total amount of CD8^+^ cells in the hippocampal region of control and PLX5622-treated WT and APP/PS1 mice (n = 6 each group). Statistical analysis was performed with the Two-way ANOVA and the exact *p* values were calculated. **e**) Representative images of granule cell and polymorph layers of the dentate gyrus in the hippocampal region of PLX5622-treated and control APP/PS1 animals. Cxcl16 signal is in white, CD8^+^ cells are in red, DAPI (blue) stains the nucleus, and Iba1 (green) is used to visualize microglia cells. Scale bar full picture: 50 µm, scale bar zoom: 10 µm. **g**) Quantitative analysis of the percentage of cells expressing Cxcl16 on the total amount of Iba1+ cells in the hippocampal region of control and PLX5622-treated APP/PS1 mice (n = 6 each group). Statistical analysis was performed with the Mann-Whitney test and the exact *p* value was calculated.

qPCR analysis on the hippocampus of 1-year-old mice revealed a significant upregulation of *Cxcl16* in samples from the APP/PS1 mouse model compared to WT animals (Two-way ANOVA, *p* = 0.0029, Šídák’s multiple comparisons test between WT CTRL and APP/PS1 CTRL, *p* = 0.0171, Fig. 3a). Similar to *Cxcl16*, gene expression analysis indicated a significant overexpression of *Cxcr6* in APP/PS1 hippocampus (Two-way ANOVA, *p* = 0.0327, Šídák’s multiple comparisons test between WT CTRL and APP/PS1 CTRL, *p* = 0.0239, Fig. 3b), supporting the role of the Cxcl16-Cxcr6 axis in APP/PS1 brain.

To test the direct involvement of microglial cells in Cxcl16-mediated T cell recruitment, we performed qPCR analysis on the hippocampus of animals treated with PLX5622, a widely used microglia depleting agent^24^. Results indicated a slight reduction of *Cxcl16* transcript (Two-way ANOVA, *p* = 0.0348, Fig. 3a), but no differences in *Cxcr6* levels (Two-way ANOVA, *p* = 0.7042, Fig. 3b), upon PLX5622-treatment. Furthermore, post-hoc comparison analysis between control and PLX5622-treated APP/PS1 animals revealed a mild but not statistical significant reduction in *Cxcl16* expression levels (Šídák’s multiple comparisons test, *p* = 0.095), implying a PLX5622-independent effect on its expression in the hippocampus of WT and APP/PS1 animals.

A weak correlation between *Cxcl16* and *Cxcr6* relative quantities was observed in control WT animals (Spearman *R* = 0.1429, *p* = 0.8028, Supplementary Figure S6a). Positive, although not significant, correlation was observed in PLX5622-treated WT and APP/PS1 mice (Spearman *R* = 0.8286, *p* = 0.0583, Supplementary Figure S6b, d). Nevertheless, the role of this chemokine-chemokine receptor axis in APP/PS1 hippocampus is supported by a strong positive correlation between *Cxcl16* and *Cxcr6* expression levels in APP/PS1 animals (Spearman *R* = 0.9429, *p* = 0.0167, Supplementary Figure S6c).

### Cxcr6^+^CD8^+^ T cell number remain unchanged upon PLX5622 treatment in the hippocampus of APP/PS1 mice

To explore the potential involvement of microglia in regulating the number of brain-resident CD8^+^Cxcr6^+^ cells, we analyzed the hippocampal region of PLX5622-treated animals.

IHC analysis of CD8^+^Cxcr6^+^ cells (Fig. 3c) revealed genotype-dependent differences between APP/PS1 and WT animals (Two-way ANOVA, *p* = 0.0002, Šídák’s multiple comparisons test between WT CTRL and APP/PS1 CTRL, *p* = 0.0007, Supplementary Fig. 7a), as previously described^20^. However, we did not observe any differences in total CD8^+^Cxcr6^+^ cell numbers (Two-way ANOVA, *p* = 0.9125, Supplementary Figure S7a) and in the percentage of Cxcr6^+^ cells among total brain CD8^+^ cells upon PLX5622 treatment (Two-way ANOVA, *p* = 0.8619, Fig. 3d).

### Pharmacological and genetic targeting of Csf1r expressing cells affects Cxcl16 expression in physiological conditions and in amyloidogenic brain tissue

To investigate the expression of *Cxcl16* in mouse brain after microglia depletion, we analyzed scRNA-seq dataset of myeloid cells isolated from the brain of control and PLX5622-treated WT animals^25^. The analysis indicated a specific cell clustering organization characterizing PLX5622-treated mice, with a clear absence of the “Macrophages” cluster (Supplementary Figure 8a, b). Violin plot indicated a statistical significant increased expression level of *Cxcl16* in “Interferon gamma signaling genes (ISG) expressing immune cells” (Wilcoxon test, *p* < 0.05) and “Neutrophils” (Wilcoxon test, *p* < 0.05) clusters of PLX5622-treated animals compared to untreated mice (Supplementary Figure 8c), pointing towards PLX5622-mediated overexpression of *Cxcl16* also in other brain-resident, or brain-infiltrating, myeloid cells.

Furthermore, we analyzed a scRNA-seq dataset of brain cells from Murno *et al*., 2024^26^. The mice included in the analysis have a deletion of the Fms intronic regulatory element (FIRE, Csf1r^ΔFIRE/ΔFIRE^, herein abbreviated as KO), which is a highly conserved region that regulates CSF1R transcript elongation in macrophages. This mutation prevents differentiation of microglia and macrophages in the skin, kidney, heart, and peritoneum^27,28^. Although no genotype-dependent statistically significant differences in *Cxcl16* expression were observed in 11–12-month-old animals (Supplementary Figure 9a-c), analysis of older mice (16–18-month-old) revealed elevated levels of Cxcl16 transcript expression in the myeloid cell compartment (including border-associated microglia (BAMs), dendritic cells (DCs) and monocytes (Mono), Wilcoxon test, *p* < 0.001), and in mural cells (Wilcoxon test, *p* < 0.0001), when compared with age-matched wild-type littermates (Csf1r^WT/WT^, referred to as WT, Supplementary Figure 9d-f).

To verify whether this compensatory expression of Cxcl16 also occurs in a brain affected by AD, we performed IHC analysis of Iba1^+^Cxcl16^+^ cells in the hippocampus of control and PLX5622-treated APP/PS1 mice (Fig. 3e). Since Cxcl16 signal was barely detectable in the brain tissue of either control and PLX5622-treated WT mice (Supplementary Figure 7b), Iba1^+^Cxcl16^+^ cells could not be quantified in WT mice. Quantification in brain tissue of APP/PS1 mice revealed an increased percentage of Cxcl16^+^ signals (white) among total brain Iba1^+^ cells (green) following PLX5622 treatment (Mann-Whitney test, *p* = 0.0087, Fig. 3f). Furthermore, we quantified the relative amount of Iba1^+^Cxcl16^+^ cells in close proximity to CD8^+^ cells (red, Fig. 3e). Therefore, we generated three-dimensional regions of interest (3D ROIs) for each channel (Supplementary Figure 7c) to measure the distance between CD8^+^ cells and Iba1^+^Cxcl16^+^ cells in x, y and z planes. By having defined a threshold of 100 μm distance, which is a measure previously reported in the literature to define T lymphocytes and Cxcl16 expressing cells proximity^29^, we found that the relative amount of Iba1^+^Cxcl16^+^ cells in close proximity with CD8^+^ cells did not change following PLX5622 treatment in APP/PS1 hippocampus (Mann-Whitney test, *p* = 0.8182, Supplementary Fig. 7d). In summary, while PLX5622 does not appear to influence the number of Iba1^+^Cxcl16^+^ cells in close proximity to CD8^+^ cells, it does lead to an increased percentage of Iba1^+^Cxcl16^+^ cells among the remaining Iba1^+^ cells.

## Discussion

The accumulation of CD8^+^ T cells in AD brain tissue is redefining our knowledge about the role of the adaptive immune system in the neurodegenerative CNS. Identifying the molecular mechanism leading to parenchymal infiltration of CD8^+^ T cells helps to explore their function in the pathological process of AD. In the present study, we assessed the role of microglial Cxcl16 in the putative recruitment of Cxcr6^+^CD8^+^ T cells to the brain of APP/PS1 animals.

A recent investigation has shown that CXCL16-CXCR6 signalling between a particular subset of monocytes (CD14^−^CD16^+^, also referred to as “nonclassical monocytes”^30)^ and CD8^+^ T cells is specific of CSF from cognitively impaired individuals. The increased availability of CXCL16 in the CSF, as well as the upregulation of CXCR6 in CD8^+^ T cells, points to this axis as a key mechanism for T cell entry into the brain^18^. Moreover, scRNA-seq analysis on West-Nile Virus (WNV) CNS-infection further supports the role of the CXCL16-CXCR6 axis in CD8^+^ T cells brain infiltration. In particular, the authors observed that anti-Cxcl16 antibody treatment prevents CD8^+^CD103^+^ T cells accumulation in the forebrain of infected animals^19^. Besides the CNS, this chemokine-chemokine receptor axis plays a critical role in the recruitment of CD8^+^ T_RM_, as observed in the airways of influenza virus-infected animals^31^.

However, the role of CXCL16-CXCR6 axis is apparently not only restricted to CD8^+^ T lymphocytes recruitment to specific tissues, but it is also involved in the accumulation of neutrophils in the CSF during pneumococcal meningitis infection^32^, in the migration of brain tumor cells^33^, in the progression of glial tumors^34^ and in breast cancer-associated brain metastasis^35^.

Our study aimed to dissect the putative role of the CXCL16-CXCR6 axis in AD-specific recruitment of CD8^+^ T cells to the brain. Indeed, qPCR analysis revealed a strong positive correlation between *Cxcr6* and *Cxcl16* expression in the hippocampus of APP/PS1 mice, pointing towards an intimate relation between these molecules in AD pathology. Taking advantage of publicly available murine scRNA-seq datasets, we confirmed that *Cxcr6* transcript is observed in lymphocytes expressing *Cd8a* and we discovered that *Cxcl16* expression characterizes brain macrophages, in particular, *Aif1* expressing cells. To prove the microglial origin of Cxcl16, we further sub-clustered “Macrophages” cells from Van Hove *et al*., 2019 and we observed that *Cxcl16* expression levels are increased in APP/PS1-associated DAMs. The increased mRNA expression levels of *Cxcl16* observed in the hippocampus of 12-month-old APP/PS1 animals are in line with the onset of CD8^+^ T cell accumulation in the brain parenchyma^6^. *Cxcl16* overexpression in the APP/PS1 hippocampus is corroborated by the upregulation of its human orthologue in different brain areas of AD-affected patients and the positive correlation of Cxcl16 protein level with Aβ and tau pathology, in other AD animal models^36^.

Interestingly, the involvement of human CXCL16 in cognitive impairment and dementia is also supported by other studies on human subjects, showing an inverse correlation between CXCL16 plasma levels and total brain volumes^37^ and a direct correlation of its serum levels with the brain presence of large atherosclerotic plaque and micro-embolic signs^38^. Moreover, increased CXCL16/Cxcl16 expression has been found in brain tissue of multiple sclerosis (MS) patients and corresponding animal models^39–41^, and in brain areas affected by ischemic insults^42^.

The AD-specific upregulation of *Cxcl16* in CPM, IRM and TM cell clusters in the dataset from Sierksma *et al*., 2020 suggests that the expression of mutant APP and PSEN1 transgenes is one of the main drivers of *Cxcl16* production. This is corroborated by the elevated Cxcl16 signals observed around Aβ plaques in representative IHC images of the APP/PS1 hippocampal area in mice. The influence of Aβ pathology in microglial *Cxcl16* expression is further supported by the statistically significant overexpression of *Cxcl16* transcript in aggregated Aβ_42_-treated BV-2 cells and monomeric Aβ_42_-treated primary microglia. Interestingly, while monomeric Aβ_42_ had no effect on sCxcl16 in BV-2 cells, we observed a statistically significant increase in sCxcl16 in the supernatant of primary microglia treated with either monomeric or aggregated Aβ_42_. This finding further supports the specific expression of Cxcl16 in microglia associated with AD pathology. Although studies indicate that the release of other cytokines from stimulated primary microglia cells might depend on Aβ_42_ conformation^43,44^, further experiments are needed to establish whether Aβ conformational state influences the expression or membrane shedding of the CXCL16 protein. These experiments could involve using *in vitro*- or *in vivo*-derived Aβ conformers (e.g. oligomers, protofibrils and fibrils), as well as inhibiting ADAM10 and ADAM17, which are involved in the proteolytic cleavage of CXCL16^45,46^.

The major genotype-related differences in *Cxcr6* expression characterized the “Effector CD8^+^ T cells” cluster from Van Hove *et al*., 2019. This observation is in line with the increased *Cxcr6* transcript levels we observed in the hippocampus of APP/PS1 animals and its co-localization with CD8^+^ T cells populating APP/PS1 mouse brains^20^. Nevertheless, besides AD-related conditions, Cxcr6^+^CD8^+^ T lymphocytes were also found to be characteristic for mixed active/inactive lesions in MS patients^47,48^, glioblastoma-affected tissues^49^ and Herpes Simplex Virus-infected trigeminal ganglia^50^. However, *Cxcr6* expression is not only restricted to CD8^+^ T cells. Indeed, our scRNA-seq analysis revealed its expression in other brain-derived CD45^+^ cells, such as “hematopoietic progenitor cells” (especially in APP/PS1 brain), “ISG expressing cells”, “memory CD4^+^ T cells”, “Natural killer cells” and “γδ-T cells”, suggesting it as a common marker for immune cells recruited to the brain.

To address the role of microglial Cxcl16 in the recruitment of Cxcr6^+^CD8^+^ T cells, we analyzed brain tissue from animals treated with PLX5622, a drug targeting and depleting Csf1r-expressing microglial cells^24^. In the hippocampus of PLX5622-treated APP/PS1 mice, we observed a mild but not statistically significant reduction of *Cxcl16* levels compared to untreated animals. Moreover, we did not achieve statistically significant changes in *Cxcr6* expression upon PLX5622 treatment. These gene expression data were validated via IHC analysis of Cxcr6^+^CD8^+^ cells in the hippocampal region from control and microglia-depleted mice. Results revealed that, while a consistent reduction of Iba1^+^ cells is evident, no changes in the amount of Cxcr6^+^CD8^+^ cells were observed in PLX5622-treated APP/PS1 animals. We would have expected microglia as the major attractant for CD8^+^ T cells, as recently demonstrated in a mouse model of tauopathy^51^, but PLX5622-mediated microglia depletion did not reduce the amount of hippocampal Cxcr6^+^CD8^+^ cells. Our results are partially supported by previous findings relative to PLX5622 treatment. Indeed, flow cytometry analysis of PLX5622-mediated microglia depletion in APP/PS1 mouse model revealed elevated total levels of CD3^+^CD8^+^ T cell in brain homogenates^12^. An increased number of CD8^+^ T cells was also observed within the brains of neurotropic hepatitis virus-infected mice, treated with PLX5622^52^. Moreover, although a reduction in *Cxcl16* transcript levels was present in the brain of PLX5622-treated WNV-infected animals, an unexpected increase in the CD8^+^ T cell count was observed^53^. The biological reason for the presence of CD8^+^ T cells in the brain of microglia-depleted animals is probably due to peripheral changes resulting from systemic PLX5622 treatment, potentially involving other chemokine-chemokine-receptor axes. Our data suggests that CD8^+^ T_RM_ cells, characterized by the expression of Cxcr6, are not affected by microglia depletion via PLX5622.

With the aim of finding a molecular reason explaining the presence of Cxcr6^+^CD8^+^ cells in the parenchyma after PLX5622 treatment, we analyzed scRNA-seq data from brain-derived myeloid cells of wild type animals treated with PLX5622. Surprisingly, we observed a striking overexpression of *Cxcl16* in various brain-infiltrating myeloid cell population following PLX5622 treatment (in particular, ISG expressing immune cells and neutrophils). These results, which partially counteract the mild reduction of *Cxcl16* observed in qPCR analysis of the hippocampi from PLX5622-treated animals, demonstrate the higher accuracy of single-cell sequencing analysis compared to bulk gene expression studies.

However, since Zhan *et al*., 2020 dataset was restricted to brain myeloid cells (CD11b^+^), we cannot exclude that Cxcl16 can be expressed also by other cells of the CNS. Indeed, CXCL16 was observed also in human astrocytes, vascular cells, and endothelial cells^46^. Furthermore, in primary murine hippocampal culture, Cxcl16 is also expressed by neuronal cells^42^. We therefore analyzed a scRNA-seq dataset comprising brain cells from mice with a genetically ablated promoter involved in Csf1r transcription. Genotype-specific differences in Cxcl16 expression were only observed in myeloid and mural cells of older animals. This suggests that the effect of compensatory Cxcl16 expression following the genetic depletion of Csfr1^+^ cells depends on the age of the animals.

To strengthen the possibility of a compensatory mechanism involving the CXCL16-CXCR6 axis occurring upon PLX5622-mediated microglia depletion also in amyloidogenic brain, we conducted IHC analysis of Cxcl16 in the hippocampal area of control and PLX5622-treated APP/PS1 mice. Although no difference in the percentage of microglial (Iba1^+^) Cxcl16^+^ cells in the proximity of CD8^+^ cells between control and PLX5622 treated mice was observed, a significant increase in the relative amount of Iba1^+^Cxcl16^+^ cells was evident upon PLX5622 treatment. This indicates that, in addition to the putative activity of other myeloid cells, PLX5622 treatment increases Cxcl16 expression in the remaining microglia cells of APP/PS1 mouse brain.

**Fig. 4:**
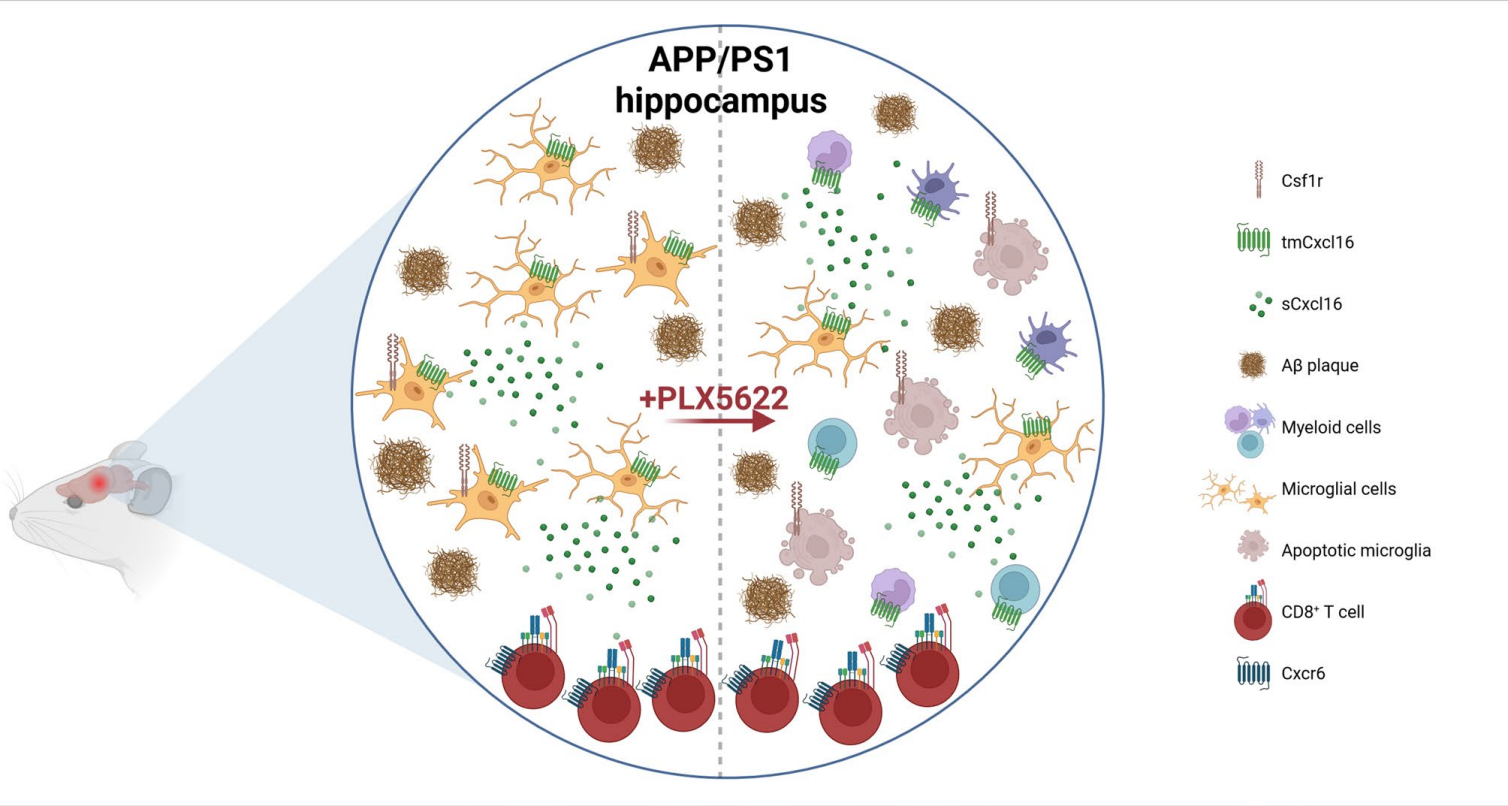
**A working hypothesis for the effect of PLX5622 in the recruitment of Cxcr6**^+^**CD8**^+^ **T cells to the brain of APP/PS1 mice.** In APP/PS1 hippocampus, Cxcl16 is mainly secreted (sCxcl16), or expressed in its transmembrane form (tmCxcl16), by microglia in the proximity of Aβ plaques, where it may act as attractant for the recruitment of Cxcr6^+^CD8^+^ T cells. Upon PLX5622 treatment, all microglia cells expressing Csf-1r undergo apoptosis and Cxcr6^+^CD8^+^ T cell levels are unaffected because due to induction of Cxcl16 expression by PLX5622-resistant microglia or by other myeloid cells populating the brain. Figure created with BioRender (biorender.com).

Our current hypothesis is that within the context of AD, PLX5622 treatment not only eliminates Csf1r-expressing microglial cells responsible for most of Cxcl16 production, but at the same time induces expression of Cxcl16 in other brain-resident (*e.g.* PLX5622-resistant microglia) or newly recruited myeloid cells, or even non-myeloid cells of the CNS. This is supported by the statistically significant increase of Cxcl16 transcript and secreted protein levels observed in both monomeric and aggregated Aβ_42_-treated BMDMs. Therefore, even upon a PLX5622-mediated depletion of microglia, other cellular sources of Cxcl16 help to maintain the population of Cxcr6^+^CD8^+^ T cells within the brain of APP/PS1 mice (Fig. 4).

### Limitations of the study

Our findings suggest a correlation between the CXCL16-CXCR6 axis and AD pathology, but it remains unknown whether modulating this axis would affect the microglia-mediated CD8^+^ T cell trafficking in the brain or influence AD progression, as recently questioned^54^. Cxcl16 has been already proposed to exert neuroprotective and anti-inflammatory activities in the brain^55,56^, pointing towards a Cxcl16-stimulation strategy for AD. Moreover, although CD8^+^ T cell ablation led to increased expression of genes related to neuronal and synapse formation^6^, recent findings suggested a protective role for brain CD8^+^ T cells and Cxcr6 in mouse AD pathogenesis^57^.

Beside our current hypothesis, the absence of any changes in Cxcr6^+^CD8^+^ cells in the brain of microglia-depleted animals could be explained by our *in vivo* experimental settings. Four weeks of PLX5622 treatment might not be sufficient to trigger a reduction of microglial-derived Cxcl16 levels and, therefore, to decrease the presence of Cxcr6^+^CD8^+^ cells in the hippocampus. Alternatively, the Cxcr6^+^CD8^+^ cells observed in the microglia-depleted brains were residing in the brain already before microglia depletion. Furthermore, sex-dependent differences in the efficiency of PLX5622 have been reported in literature^58,59^. Consequently, the potential impact of gender bias in the microglia Cxcl16-mediated recruitment of Cxcr6^+^CD8^+^ cells in the brain of treated animals cannot be excluded. The limited number of animals in each experimental group from our PLX5622 *in vivo* treatment does not allow us to consider this factor in the present study.

While the potential for phenotypic changes in brain-resident T cells following PLX5622 treatment cannot be discounted, the present study was not designed to investigate the immunological signature of these lymphocytes following microglia depletion in AD. A substantial body of research suggests that PLX5622-dependent depletion of microglia exerts an influence in the quantity and phenotype of T cells in various disease contexts, including viral infections, MS and the aging process^52,53,60–64^. Consequently, further single-cell experiments on an AD transgenic mouse model are required to ascertain whether PLX5622-mediated depletion of microglia affects T cell activation status in AD.

Furthermore, we do not dismiss the potential role of other chemokine-chemokine receptor axis and different cell types, beyond microglia, in the recruitment of CD8^+^ T lymphocytes to the brain. Recently, the Cxcl13-Cxcr5 axis has been demonstrated to be essential in the recruitment of CD8^+^ T cells from injured neurons^65^. However, this axis is also involved in recruiting other lymphocytic cells to the CSF^66,67^, suggesting a pleiotropic function of Cxcl13-Cxcr5 communication in disease and pathology. Interestingly, a recent neuronal-microglia-CD8^+^ T cells co-culture system revealed key roles for the C-X-C motif chemokine ligand 10 (CXCL10) and its receptor, CXCR3, in regulating T cell infiltration in cell cultures derived from AD patients^68^. On the other hand, the CXCL16-CXCR6 axis could play multiple roles in the calls-to-arms of different peripheral cells, other than CD8^+^ T lymphocytes, towards AD-affected brain tissue.

## Conclusions

Understanding the molecular mechanism involved in the accumulation of adaptive immune cells in neurodegenerative-affected brains is essential to further explore their functional role in disease progression. Here, we hypothesize a putative involvement of the CXCL16-CXCR6 axis in AD pathology. Although our data suggest an active contribution of microglial cells in Cxcl16 production in AD, IHC analysis of the hippocampi from PLX5622-treated APP/PS1 animals revealed no changes in the recruitment of CD8^+^Cxcr6^+^ cells to the brain parenchyma.

Alternative models of microglia depletion or modulation of the CXCL16-CXCR6 axis in AD pathology could help to understand the contribution of CXCL16-CXCR6 immune axis in the recruitment of CD8^+^ T cells to the brain.

## Methods

### ScRNA-seq datasets analysis

ScRNA-seq datasets were analyzed with Seurat package (version 5.0.1), following the guided clustering tutorial . Data from Van Hove *et al*., 2019^21^ and Zhan *et al*., 2020^25^ were retrieved using the *Read10X* function, while the *read_tsv* function was used to access count data and metadata from Sierksma *et al*., 2020^23^. With *CreateSeuratObject*, we generated the related Seurat objects. In Van Hove *et al*., 2019, cells from 16-month-old wild type (WT) and APP/PS1 mice (6945 total cells) were split, according to genotype, and then cells from 16-month-old APP/PS1 animals were merged together with 9-month-old animals (7831 total cells). Sierksma *et al*., 2020 dataset was subset only for 11-month-old mice expressing APP Swedish and PSEN1-L166P transgenes (herein abbreviated as APP/PS1-L166P) and respective WT animals (3821 total cells). Zhan *et al*., 2020 dataset included three biological replicates which were merged together in two groups (namely PLX5622 and CTRL, 26054 total cells). The quality control metrics of each dataset were visualized with violin plot. To exclude empty droplets with few detected genes and multiplets with aberrantly high gene count, datasets were subdivided to contain more than 200 and less than 5,000 unique genes (nFeature_RNA) and less than 30,000 UMI counts (nCount_RNA). A threshold of 5% was set for the amount of mitochondrial genes. We normalized the feature expression levels by the total expression for each cell with the *LogNormalize* function. To calculate a subset of features exhibiting high cell-to-cell variation in the datasets, the *FindVariableFeatures* function was used. As a standard pre-processing step prior to dimensional reduction, the *ScaleData* function linearly transformed the expression of each gene. Principal Component Analysis (PCA) was run on the scaled highly variable features. After ranking principal components using the percentage of variance revealed by the *ElbowPlot* function, cell clustering was performed with the *FindNeighbors* function, based on the first 30 principal components (PCs), and the *FindClusters* function with resolution set as 1. Dimensional reduction was performed using the *RunUMAP* function accounting 30 PCs. Uniform Manifold Approximation and Projection (UMAP) visualization was plotted using DimPlot, with “umap” as reduction option. *FeaturePlot* was used to present features expression level at single cell level on UMAP, displaying cluster-specific expression genes. Automated cell type annotation^22^ was applied to Van Hove *et al*., 2019 and Zhan *et al*., 2020 datasets. With the *auto_detect_tissue_type* function, “immune system” has been selected as the tissue representing the datasets with the highest probability. The assigned clusters were visualized by grouping them according to the newly generated “clustomclassif” metadata identity. After automated cell type annotation, the “Unknown” cluster of myeloid cells from the Zhan *et al*., 2020 dataset was removed for an ease of representation. Total cell clusters proportion plots were generated with the *RColorBrewer* package. The table representing the percentage of cells expressing determined transcripts from the Van Hove *et al*., 2019 dataset, was generated using the output from *Percent_Expressing* and *PrctCellExpringGene* functions. To dissect the “Macrophages” cell cluster from the Van Hove *et al*., 2019 dataset, we subset the original clusters and we used the *FindAllMarkers* function to rename them according to established scRNA-seq microglia/macrophages markers^69–71^. We created the heat map of the clusters representing “Macrophages” cells, showing the main four differentially expressed genes for each cluster using the *DoHeatmap* function. Microglia cells from the Sierksma *et al*., 2020 dataset were visualized using features from the already established “cell.states” metadata identity. *VlnPlot* function was used to represent the expression levels of selected transcript grouping data by the cell types and splitting for either genotype (Van Hove *et al*., 2019 and Sierksma *et al*., 2020 datasets) or treatment (Zhan *et al*., 2020 dataset).

The scRNA-seq dataset from Munro *et al*., 2024^26^ was analyzed using the SingleCellExperiment package (version 1.31.1), in accordance with the user manual. The dataset included three biological replicates of 11–12- and 16–18-month-old *Csf1r*^WT/WT^ (referred to as WT) and *Csf1r*^ΔFIRE/ΔFIRE^ (herein abbreviated as KO) mice. Data were retrieved using *readRDS* function. The SingleCellExperiment object was visualized using genotype and features from the established cell types metadata (*i.e.* “clusters_named”) as variables via *plotReducedDim* function from scater package. Finally, *ggcells* function was used to plot the expression levels of the Cxcl16, grouping the data by the cell types and splitting by genotype.

### Animals

APP Swedish and PSEN1-dE9 mice, expressing a chimeric mouse/human mutant amyloid precursor protein (Mo/HuAPP695swe) and a mutant human presenilin 1 (and PSEN1-dE9), both directed to CNS neurons under the prion protein promoter (available by Jackson Laboratory, http://www.jax.org/strain/005864, RRID: MMRRC_034832-JAX), were used. Mice were housed at the Paracelsus Medical University Salzburg under standard conditions at a temperature of 22 °C and a 12-h light/dark cycle with *ad libitum* access to standard food and water. Local ethical committees (BMWFW-66.019/0032-WF/V/3b/2016) approved animal care, handling, genotyping, and experiments.

For this study, 12-month-old female and male animals were used and treated for 28 days with PLX5622 chow^12^. Age-matched non-transgenic mice, derived from the breeding of APP Swedish PS1 dE9 (herein abbreviated as APP/PS1) were used as control animals (herein referred as WT). All animals were adapted to control chow two weeks before introducing the PLX5622 chow. Thus, after computer based randomisation, four experimental groups were generated: WT and APP/PS1 mice, which received control chow (WT CTRL and APP/PS1 CTRL, respectively), together with WT and APP/PS1 mice, which received the PLX5622 chow for a total of 28 days (WT PLX5622 and APP/PS1 PLX5622, respectively). The researchers experimenters could not be blinded because the chow were added to the cages to which the pre-defined different animal groups belonged to. No inclusion and exclusion criteria were defined a priori.

### Tissue collection

After four weeks of treatment, mice were anesthetized by intraperitoneal injection of a xylazine (5.36 mg/mL final, Xylapan 20 mg/mL, Vetoquino Österreich GmbH, Z.-Nr. 8–00359), ketamine (20.5 mg/mL final, Ketamidor 100 mg/mL, VetViva Richter GmbH, Z.-Nr. 8–01141),

and acepromazine maleate (0.27 mg/mL final, Vanastress 10 mg/mL, VANA GmbH, E1123-S.1216, Z.-Nr. 8–00442) mixture. Afterwards, their thoracic cavity was opened with an incision caudal to the sternum. Animals were manually perfused through the left ventricle with 1× ice cold Hank`s Balanced Salt Solution (Thermo Fisher Scientific, #14175-053) containing 15 mM HEPES (Thermo Fisher Scientific, #15630-106) and 0.5% glucose (Sigma-Aldrich, #G7021) to rinse out the blood. Mice were decapitated and brains were extracted from the skull. Hippocampus from one brain hemisphere was immediately transferred to RNAlater^®^ (Sigma-Aldrich, #R0901) and stored at −80 °C. The other brain hemisphere was immersed in 4% paraformaldehyde (in 0.1 M sodium phosphate solution, pH = 7.4, Sigma-Aldrich, #S9390) for fixation over night before being washed in 1× PBS and transferred into 30% sucrose (AppliChem, #A2211) for cryoprotection. When fully soaked with sucrose, brain hemispheres were cut in 40 μm slices on dry ice using a sliding microtome (Leica) dividing them in representative tenths of the brain. Sections were stored at −20 °C in cryoprotectant solution (Ethylene Glycol, Sigma-Aldrich, #100949, Glycerol, AppliChem, #131339.1211, 0.1 M phosphate buffer, Sigma-Aldrich, #D8537, in a 1:1:2 volume ratio).

### BV-2 cells culture

Murine microglial BV-2 cells (originally obtained from Banca Biologica and Cell Factory, IRCCS Azienda Ospedaliera Universitaria San Martino, Genova, Italy, RRID: CVCL_0182) were maintained in Dulbecco’s Modified Eagle’s Medium (DMEM) with high glucose (Thermo Fisher Scientific, #11995065) and DMEM low glucose (Sigma-Aldrich, #FG0415) in a 1:1 ratio, supplemented with 10% fetal bovine serum (FBS, Thermo Fisher Scientific, #A5256701) and Penicillin-Streptomycin (Pan Biotech, # P06-07100), under standard culture conditions (95% relative humidity with 5% CO_2_, at 37 °C).

### Primary microglia isolation and maintenance

The isolation of primary microglial cells was achieved using P0-P2 pups of the C57BL/6J strain, following established protocols^72–75^. The animal breeding, handling, genotyping, and procedures were approved by local authorities (BMBFW-66.019/0011-WF/V/3b/2016 and BMBFW: 2020 − 0.827.682). Following decapitation, the brains were extracted from the skull and placed in DMEM with the addition of 10% FBS and Penicillin-Streptomycin (henceforth referred to as ‘glia medium’). After the removal of cerebellum and olfactory bulbs, the forebrains were divided in the right and left hemispheres, and the meninges were carefully removed to prevent fibroblasts growth in the culture. The tissues were placed in 50 mL Falcon tubes containing glia medium. The cell suspension was passed through a 100 μm cell strainer (Corning, #734–2762) by means of gentle trituration with a 5 mL pipette and a serological glass pipette, in order to remove any larger debris. The cell suspension from three forebrains was seeded in T75 cell culture flasks (TPP, #90076) that had been coated with 5 µg/mL poly-D-lysine (PDL) (EMD Millipore, #A-003-E in H_2_O). The cells were cultivated in glia medium in accordance with standard culture conditions. The following day, and subsequently every fourth day, the medium was replaced while the remaining media was collected, and filtered sterile with 22 μm syringe filter (TPP, #99722) and used as “conditioned” media. In the context of each media change, the new media was supplemented with 50% of the fresh glia media and 50% of conditioned media. Following a 14-day culture period, a confluent layer of mixed glia cells was observed, comprising astrocytes, oligodendrocytes, ependymal cells, and microglia. In order to dislodge the loosely adherent microglia from the confluent cell layer, the culture flasks were subjected to shaking process (“shake-off”) at a frequency of 110 rpm for a duration up to 90 min at 37 °C. Afterwards, the supernatant containing microglia cells was used for further experiments, with the cells being maintained under standard culture conditions. The culture of mixed glia could be sustained for a period of up to five “shake-offs” by the incorporation of a mixture of fresh and conditioned media.

### Establishment of mouse bone marrow-derived macrophages

BMDMs were prepared and cultured from C57BL/6J mice , using established procedures adapted from published protocols^76,77^. In brief, following cervical dislocation, any remaining muscle tissue and tendons on the tibia and femur were cleared with gauze. The bone marrow progenitor cells were then flushed with ice cold 1× PBS and passed through the 100 μm and the 70 μm cell strainers (Corning, #734–2762 and #734–2761). The cells were then centrifuged at 250×g, 4 °C for 5 min. Red blood cells were removed with Red cell lysis buffer (from Adult Brain Dissociation Kit, Miltenyi Biotec, #130-107-677). The cell suspension was then centrifuged again (250×g, 4 °C for 5 min). The cells were seeded in glia medium containing 10 ng/mL macrophage colony-stimulating factor (M-CSF, Peprotech, #315-02). The cultures were maintained for 7 days with a media change on day 4 under standard culture conditions.

### Flow cytometry

To monitor the purity of the primary murine culture, microglia cells after “shake-off” and BMDMs were analyzed by flow cytometry. 1*10^5^ cells were washed with 1 mL 1× PBS, resuspended with 1 mL of flow buffer (2% (w/v) bovine serum albumin (BSA), Sigma-Aldrich, #A9647, and 200 mM ethylenediaminetetraacetic acid, Sigma-Aldrich, #E1644, in PBS) and stained with a 1:10,000 dilution of Live/Dead 488 viability dye (Thermo Fisher Scientific, # L34969). The cells were incubated at room temperature (RT) in the dark for 30 min. After washing with flow buffer, Mouse BD Fc Block™ (1:100, BD Pharmingen™ Purified Rat Anti-Mouse CD16/CD32, BD Biosciences, #553142) was added and the cells were incubated again at RT in the dark for 5 minutes. This was followed by immunostaining with anti-rat CD11b APC (1:50, clone M1/70; BioLegend, #101211) and anti-rat F4/80 PerCP-Cyanine5.5 (1:75, clone BM8, Invitrogen™, #45-4801-80) for 30 min at 4 °C in the dark. After washing with 2 mL flow buffer, the cells were resuspended in 300 µL flow buffer and analyzed using a BD Accuri C6 flow cytometer (BD Biosciences). The data were analyzed using BD Accuri C6 software (BD Biosciences).

### Cell culture experiments

BV-2 cells were seeded at a density of 2.5*10^4^ cells per well in a 6-well tissue culture plate (TPP, #92406). Two days later, the cells were treated with either 1 µM of monomeric or aggregated (after an overnight incubation at 37 °C) human Aβ_42_ (rPeptide, #A-1002-1), for 24 h in FBS-depleted medium, in duplicate. The controls received either medium only (herein referred as “Medium”) or the solution used to dissolve Aβ_42_ peptide (1% NH_4_OH, herein referred as “Vehicle”, corresponding to 0.0045% NH_4_OH, Sigma-Aldrich, #221228). For the primary microglia experiments, 6.6 *10^4^ cells per well were seeded in a 96-well tissue culture plate (TPP, #92696). BMDMs were seeded at a density of 1.5*10^4^ cells per well in a 96-well tissue culture plate (Nunc UpCell Surface, ThermoFisher, #174897). One day (for primary microglia) or 7 days (for BMDMs) after seeding, cells were treated with Medium, Vehicle, 1 µM of monomeric or aggregated human Aβ_42_, or 1 µM of scrambled human Aβ_42_ sequence (rPeptide, #A-1004-1).

### RNA isolation and reverse transcription

BV-2 cells were lysed in 500 µL TRI^®^ Reagent (Sigma-Aldrich, #T9424) and subsequently pooled with the respective technical duplicate well for a total volume of 1 mL. Brain tissue was homogenized in 200 µL TRI^®^ Reagent with pellet pestles (Bartelt, #7.620842) and 800 µL were added after the homogenization step. For phase separation, 150 µL of 1-bromo-3-chloropropane (Sigma-Aldrich, #B9673) was added, vortexed and centrifuged (12,000×g for 15 min, at 4 °C). After transferring the aqueous phase into a new tube, 1 µL GlycoBlue™ (Thermo Fisher Scientific, #AM9516) and 500 µL 2-Propanol (Sigma-Aldrich, #1096342511) were added and, after vortexing, the samples were centrifuged (12,000×g for 10 min, at 4 °C). The pellet was washed with 1 mL 75% ethanol, dried and re-suspended in 30 µL RNase-free water (pre-warmed to 55 °C, from Promega, #A6102). Total RNA concentrations were determined with a NanoVue plus (GE Healthcare). cDNA was synthesized using GoScript Reverse Transcriptase Mix including a 1:1 ratio of Oligo(dT) (Promega, #A2790) and random primers (Promega, #A2800).

Gene expression analysis of primary murine microglia and BMDMs was performed using the TaqMan™ Fast Advanced Cells-to-CT™ Kit (Thermo Fisher Scientific, #A35377), following the manufacturer’s instructions up to the stage of synthesising cDNA.

### qPCR

Quantitative gene expression analysis (qPCR) was performed using TaqMan real time-PCR technology. Technical duplicates containing 10 ng (for hippocampal tissue) or 7.5 ng (for BV-2 cells) of cDNA were amplified with the GoTaq Probe qPCR Master Mix (Promega, #A6102) using a two-step cycling protocol (95 °C for 15 s, 60 °C for 60 s; 40 cycles using Bio-Rad CFX 96 Cycler) in LabQ 96-well PCR plate (LabShop Online, #PS3441-00 C), sealed with Real-Time PCR plate Sealing Film (LabShop Online, #PSPETST100). 2 µL of the Cells-to-CT reverse transcription solution from primary murine microglia and BMDMs were used. The following validated exon-spanning gene expression assays were employed: G6pdx (Integrated DNA Technologies, Mm.PT.58.13826440) and Ywhaz (Integrated DNA Technologies, Mm.PT.39a.22214831), as validated candidate housekeepers, Cxcl16 (Integrated DNA Technologies, Mm.PT.56a.42520449), Cxcr6 (Integrated DNA Technologies, Mm.PT.58.8341778), and Tnf (Thermofisher, Mm00443258_m1) for target gene analysis.

qPCR analysis was performed for cDNA from hippocampus of twenty-four animals (*n* = 6 WT CTRL; *n* = 6 WT PLX5622; *n* = 6 APP/PS1 CTRL; *n* = 6 APP/PS1 PLX5622) and from five independent biological experiments on BV-2 cells (*n* = 5 Medium-; *n* = 5 Vehicle-; *n* = 5 1 µM monomeric Aβ_42_-; *n* = 5 1 µM aggregated Aβ_42_-treated cells), on primary murine microglia and BMDMs (*n* = 5 Medium-; *n* = 5 Vehicle-; *n* = 5 1 µM scramble Aβ_42_-; *n* = 5 1 µM monomeric Aβ_42_-; *n* = 5 1 µM aggregated Aβ_42_-treated cells). The mRNA relative quantities was calculated with a modified version of 2^−ΔΔCT^ method^78^ taking into account the efficiency (E) of each exon-spanning gene expression assay (target genes: *Cxcr6*,* Cxcl16*, *Tnf*; reference genes: *G6pdx* and *Ywhaz*), using the following Eq. (1):

$$\begin{aligned} Relative~quantities & = 2^{{( - \Delta \Delta C_{T} )}} \\ & = 2^{{ - \left( {\Delta C_{T} ~\left( {test} \right)~{-}~\Delta C_{T} ~\left( {calibrator} \right)} \right)~}} = 2^{{ - ~\Delta C_{T} ~\left( {test} \right)~ + ~\Delta C_{T} ~\left( {calibrator} \right)~}} \\ & = 2^{{\Delta C_{T} ~\left( {calibrator} \right) - ~\Delta C_{T} ~\left( {test} \right)~}} = \frac{{2^{{\Delta C_{T} ~\left( {calibrator} \right)~}} ~~}}{{2^{{\Delta C_{T} ~\left( {test} \right)}} }} \\ & = \frac{{2^{{C_{T} ~\left( {target~gene,~calibrator} \right)~ - ~C_{T} ~\left( {reference~gene,~calibrator} \right)}} ~~}}{{2^{{C_{T}~\left( {target~gene,~test} \right)~{-}~C_{T} ~\left( {reference~gene,~test} \right)}} }} \\ & = \frac{{2^{{C_{T} ~\left( {target~gene,~calibrator} \right)~ - ~C_{T} ~\left( {reference~gene,~calibrator} \right)}} ~~}}{{2^{{C_{T} ~\left( {target~gene,~test} \right)~{-}~C_{T} ~\left( {reference~gene,~test} \right)}} }} \\ & = \frac{{\frac{{E_{{target~gene}}^{{C_{T} ~\left( {target~gene,~calibrator} \right)}} }}{{E_{{reference~gene}}^{{C_{T} ~\left( {reference~gene,~calibrator} \right)}} }}}}{{\frac{{E_{{target~gene}}^{{C_{T} ~\left( {target~gene,~test} \right)}} }}{{E_{{reference~gene}}^{{C_{T} ~\left( {reference~gene,~test} \right)}} }}}} \\ \end{aligned}$$

Firstly, we calculated the efficiency of each gene expression assays using titration of pooled sample from cDNA of WT and APP/PS1 brains and of BV-2, murine primary microglia and BMDM cells. Then, we calculated the mean E _reference gene_^CT (reference gene)^ of *G6pdx* and *Ywhaz* for each sample. The relative quantities of *Cxcr6* and *Cxcl16* expression levels in the brain tissue were obtained dividing the average E _target gene_^CT (target gene, calibrator)^/E _reference gene_^CT (reference gene, calibrator)^ of the “calibrator” WT control samples (*n* = 6) by the E _target gene_^CT (target gene, test)^/E _reference gene_^CT (reference gene, test)^ of each sample. For the relative quantities of *Cxcl16* and *Tnf* in the cell cultures (*i.e.* BV-2 cells, murine primary microglia and BMDM), the average E _target gene_^CT (target gene, calibrator)^/E _reference gene_^CT (reference gene, calibrator)^ of the “calibrator” medium-treated cells (*n* = 5) by the E _target gene_^CT (target gene, test)^/E _reference gene_^CT (reference gene, test)^ of vehicle- and Aβ_42_-treated cells. To make the plots easier to consult, avoiding negative data points, values of relative quantities smaller than 1 were not converted using the equation − 1/relative quantities.

### Western blot

2 mL of FBS-depleted conditioned medium from technical duplicates of BV-2 cells was centrifuged at 300×g for 5 min. The supernatants were then filtered (0.22 μm, TPP, #99722) to remove cellular debris. Proteins were precipitated with ice-cold acetone after 1 h at −20 °C and followed by centrifugation (15,000×g for 10 min, at 4 °C). The residual protein pellets were resuspended in 30 µL ddH_2_O, and their concentration was determined using the Bicinchoninic acid method by measuring absorbance at 562 nm on a microplate reader (Mithras LB 940, Berthold Technologies GmbH & Co.KG). 4× loading buffer (125 mM Tris pH 6.8, Fisher Scientific, #BP1521, 6% SDS, AppliChem, #A2572, 20% Glycerol, 10% β-Mercaptoethanol, Sigma-Aldrich, #444203 and 0.002% Bromophenol blue, Supelco, #1.08122) was added to 60 µg of total proteins from cells supernatants in 1:4 ratio and subsequently boiled 7 min at 95 °C. 2 µg of monomeric or aggregated Aβ_42_ were used to check their aggregation status in WB. 20 µg of total protein from BV-2 cell lysate were prepared after lysis with ice-cold RIPA buffer (20 mM Tris, pH 7.5, Fisher Scientific, #BP1521, 100 mM NaCl, VWR, #27810.364, 1% Triton ™ X-100, Sigma-Aldrich, #T9284 0.5% sodium deoxycholate, Sigma-Aldrich, #D6750, 0.1% SDS).

Samples were loaded onto 4–20% Mini-PROTEAN TGX Stain-free Gel (Bio-Rad, #4568096) and separated by electrophoresis with PowerPC^®^ Basic (Bio-Rad). The gels were transferred to PVDF membrane using the Trans-Blot^®^ Turbo Transfer pack (Bio-Rad). After stain-free signals acquisition, membranes were incubated for 1 h in 5% milk in 1× TSB-T (0.05% Tween^®^ 20, Sigma-Aldrich, #P1379, in 1× TBS) to block unspecific binding sites, and subsequently incubated overnight at 4 °C with anti-Cxcl16 antibody (1:350, Antibodies–online.com, #AA 85–200) or anti-Aβ (1:1,000, clone 6E10, Covance, #SIG-39300). After three washings in 1× TBS-T, membranes were then incubated for 1 h with goat anti-rabbit HRP secondary antibody (1:10,000, Cell Signalling Technology, #7074) or goat anti-mouse HRP secondary antibody (1:10,000, Life Tech, #A10677). Reactions were visualized by chemiluminescence on ChemiDoc™ MP Imaging System (Bio-Rad) using Clarity™ Western ECL substrate (Bio-Rad, #1705061). Densitometric analysis of sCxcl16 bands was carried out using ImageLab software (Bio-Rad) and normalized with the signals from the corresponding bands of the stain-free blot.

### ELISA

Analysis of sCxcl16 in the supernatant from primary microglia and BMDMs was performed using a commercial mouse Cxcl16 ELISA Kit (Thermo Fisher Scientific, #EMCXCL16), according to the manufacturer’s instructions. The cell supernatant samples (stored at −80 °C until following collection) were diluted 1:10 for the analysis. Absorbance at 450 nm was measured using a microplate reader (Mithras LB 940, Berthold Technologies GmbH & Co.KG). The resulting values were plotted on a standard curve, prepared with serial dilution of recombinant mouse Cxcl16, to calculate the sCxcl16 concentration (pg/mL) in the cell supernatants.

### Fluorescence IHC

IHC of mouse tissue was performed on free-floating sections as previously described^6,20^. Briefly, antigen retrieval was performed depending on the used primary antibody by steaming the sections for 15 min in 1× citrate buffer (pH 6.0, Sigma-Aldrich., #C9999). To block unspecific binding sites, slices were incubated for 1 h in blocking solution (1% BSA, Sigma-Aldrich, # A9647, 0.2% fish skin gelatine, Sigma-Aldrich, #G7765, 0.1% Tween^®^ 20 in 1× PBS). Brain slices were incubated on a shaker overnight at RT with the following primary antibodies: rat anti-CD8a (1:100, Invitrogen, #14–0195-82), rabbit anti-Cxcr6 (1:500, Thermofisher, #PA5-79117), goat anti-Iba1 (1:500, Abcam, #ab5076), rabbit anti-Cxcl16 (1:100, antibodies.online, #ABIN686572). Sections were extensively washed in 1× PBS and incubated for 4 h at RT in appropriate fluorescent-labelled secondary antibodies: donkey anti-goat Alexa 488 (Invitrogen, #A11055), donkey anti-rat Rhodamine Red (Szabo-Scandic, #JAC712295150), donkey anti-goat Rhodamine Red (Jackson, 108758), donkey anti-rabbit Alexa 647 (Invitrogen, #A31573) (all diluted 1:500), DAPI (1 mg/ml, 1:2000, Sigma-Aldrich, #D9542), for nucleus counterstaining and Amytracker 520 (1:1,200, Biozol, #EBB-A520) for Aβ plaques staining. The sections were extensively washed in 1× PBS and mounted onto microscope glass slides (Superfrost™ Plus, Thermo Fischer Scientific, #22037246). Brain sections were cover slipped semidry in ProLong Gold antifade mountant (Thermo Fischer Scientific, # P36930).

### Confocal microscopy and image processing

For fluorescence imaging, the confocal laser scanning microscope LSM 710 from Zeiss was used. Images were taken with the ZEN 2011 SP3 software (black edition, Zeiss). For representative pictures of CD8^+^Cxcr6^+^, Iba1^+^ cells, or Cxcl16^+^ signals, were obtained as confocal z stacks in ×40 or ×63 oil magnification of granule cell and polymorph layers of the dentate gyrus in hippocampal region. Images were combined to merged maximum intensity projections and edited as well as processed with the ImageJ/Fiji software (version 1.53q) and Microsoft PowerPoint.

For quantification of Cxcr6^+^CD8^+^ and Cxcl16^+^Iba1^+^ cells were counted in at least nine images (320 × 320 μm) from three to four different hippocampal sections (including the dentate gyrus and Cornu Ammonis 1 (CA1) to CA3 regions), with three to five images per section, using ImageJ’s cell counter plugin. This resulted in an average of ∼50 analyzed cells per animal.

An ImageJ macro was used to quantify the percentage of Cxcl16^+^Iba1^+^ cells and analyse their distance (in 3D) to CD8^+^ cells. Firstly, scale bar was converted to pixels per µm (using a conversion factor of 2.8927 px/µm). Brightness and contrast adjustments were performed differently for each channel. After filtering the images with the Gaussian Blur 3D plugin, the 3D object counter plugin provided a list of voxels for each channel. The 3D Manager plugin then automatically selected segments for each channel (a minimum of 20 for Iba1 channel, a minimum of 10 for CD8 channel, a minimum of 10 for Cxcl16 channel and a maximum of 255 for every channel). 3D ROIs corresponding to CD8^+^ cells were selected manually. The 3D ROIs for Iba1 and Cxcl16 were automatically identified by setting a voxel volume greater than 500 for the former and a voxel volume ranging from 4 to 300 for the latter. Finally, the “Measure” extension of the 3D Manager plugin was used to automatically compute the distances between all objects.

Subsequent analysis was conducted in R (version 4.4.2) using the dplyr package. ImageJ output files were retrieved using the *read.csv* function. Firstly, Iba1 cells located within a 15 μm border-to-border distance of Cxcl16 ROIs (considered to be double-positive Iba1^+^Cxcl16^+^ cells) was quantified. Then the percentages over the number of total Iba1^+^ ROIs was calculated. Their distance from CD8^+^ ROIs was then measured. The analysis included the cells with a border-to-border distance lower than 100 μm, as recently defined for measuring the proximity of lymphocytes to myeloid cells expressing Cxcl16 in the brain tissue^29^.

### Statistics

All graphing and statistical analysis were performed using GraphPad Prism 9 or R. scRNA-seq data represented in violin plots were analyzed using *stat_compare_means* function with Wilcoxon test. Data from qPCR and IHC analysis were tested for normal distribution with D’Agostino & Pearson test. To identify outliers, the ROUT method was applied. For hippocampal gene expression of *Cxcr6* and *Cxcl16* transcripts and for IHC Cxcr6^+^CD8^+^ cells quantification, data were analyzed with Two-way ANOVA and Šídák’s multiple comparisons test. Linear regression was performed for *Cxcr6* and *Cxcl16* transcript levels and tested with Spearman R correlation analysis. For Cxcl16 gene expression, WB and ELISA analysis in *in vitro* cell culture experiments, Friedman test with Dunn`s multiple comparison (between vehicle and monomeric or aggregated Aβ_42_-treated cells in BV-2, and between scrambled Aβ_42_- and monomeric or aggregated Aβ_42_-treated cells in primary microglia and BMDMs) was used. The IHC quantification of Cxcl16^+^Iba1^+^ cells was assessed with Mann-Whitney test. The level of statistical significance was set at 0.05 for all analyses. For scRNA-seq data analysis, *p* values were reported as < 0.05, < 0.01, < 0.001 or < 0.0001. *p* values were not indicated for non-statistically significant differences. For all the other analyses, *p* values representing statistical significant differences in Two-way ANOVA, in Friedman, in Mann-Whitney and in multiple comparison tests are shown in the plot. The data were represented as individual values alone or together with the mean and standard error of the mean.

## Supplementary Information

Below is the link to the electronic supplementary material.

Supplementary Material 1

## Data Availability

The mouse scRNA-seq datasets used in this study derived from Brain Immune Atlas website (https://www.brainimmuneatlas.org/index.php) and the Gene Expression Omnibus repository, under the accession numbers GSE128855, GSE142267, GSE150169, and GSE267545. Any additional data supporting the findings of this study are included in the article and related supplementary information files.

## Declarations

### Approval for animal experiments

All animal experiments were approved by the Austrian Ministry of Science and Research (BMBFW-66.019/0011-WF/V/3b/2016 and BMBFW: 2020 − 0.827.682) and were performed in accordance with relevant named guidelines and regulations. Furthermore, the authors complied with the ARRIVE guidelines, as the “ARRIVE 10 essentials” are described in details in the “Methods” section.
