# Publisher Correction: The involvement of microglia and the CXCL16-CXCR6 axis in the recruitment of CD8^+^ T cells to an amyloidogenic mouse brain

**DOI:** 10.1038/s41598-026-51926-9

**Published:** 2026-06-24

**Authors:** Marco Zattoni, Sabine Bernegger, Sofia Weinbender, Barbara Altendorfer, Heike Mrowetz, Ariane Benedetti, Rodolphe Poupardin, Michael Stefan Unger, Ludwig Aigner

**Affiliations:** 1 Institute of Molecular Regenerative Medicine , Paracelsus Medical University, Salzburg, Austria; 2 Institute of Experimental Neuroregeneration , Paracelsus Medical University, Salzburg, Austria; 3 Institute of Experimental and Clinical Cell Therapy , Paracelsus Medical University, Salzburg, Austria; 4 Austrian Cluster for Tissue Regeneration, Vienna, Austria

Correction to: *Scientific Reports* 10.1038/s41598-025-22137-5, published online 31 October 2025

The original version of this Article contained errors where the legends of the figures were omitted.

The original Article has been corrected.

