# Supplementary material for: The involvement of microglia and the CXCL16-CXCR6 axis in the recruitment of CD8+ T cells to an amyloidogenic mouse brain
Source: Sci Rep. 2025 Oct 31;15:38221. doi: 10.1038/s41598-025-22137-5 (PMC12578786; doi:10.1038/s41598-025-22137-5)
Supplement: Supplementary file 1 — Supplementary Material 1 [file 41598_2025_22137_MOESM1_ESM.pdf]

Supplementary Information

Title

The involvement of microglia and the CXCL16-CXCR6 axis in the recruitment of $CD8^+$ T cells to an amyloidogenic mouse brain

Authors

Marco Zattoni[1, *, #], Sabine Bernegger[1, #], Sofia Weinbender[1], Barbara Altendorfer[1], Heike Mrowetz[1], Ariane Benedetti[2], Rodolphe Poupardin[3], Michael Stefan Unger[1], Ludwig Aigner[1, 4, *]

Affiliations

[1] Institute of Molecular Regenerative Medicine, Paracelsus Medical University, Salzburg, Austria

[2] Institute of Experimental Neuroregeneration, Paracelsus Medical University, Salzburg, Austria

[3] Institute of Experimental and Clinical Cell Therapy, Paracelsus Medical University, Salzburg, Austria

[4] Austrian Cluster for Tissue Regeneration, Austria

* *Corresponding authors*: Ludwig Aigner; Marco Zattoni

# Equally contributing first authors: Marco Zattoni, Sabine Bernegger

	WT	APP/PS1
Total counts	844.63	2894.24
% Total Cell Proportion	16.83	36.08
% Macrophages	3.27	6.05
% Effector CD8+ T cells	78.99	83.16
% Memory CD8+ T cells	28.95	27.98
% Memory CD4+ T cells	84.78	81.88
% Naive B cells	2.73	4.49
% Neutrophils	2.18	7.32
% ISG expressing immune cells	47.83	69.83
% Natural killer cells	51.30	54.55
% Erythroid like and erythroid precursor cells	0.00	10.68
% γδ T cells	100.00	91.40
% Progenitor cells	17.31	54.39
% Pre B cells	2.60	7.69
% Myeloid Dendritic cells	2.08	10.26
% Basophils	31.58	50.00

Supplementary Table S1. *Cxcr6* expression counts and percentages in scRNA-seq brain-derived $CD45^+$ cells. The table lists the total counts, the total cells proportions and the percentages of expression of *Cxcr6* in the different cell clusters automatically identified in Van Hove *et al*., 2019 dataset, between WT and APP/PS1 brain-derived $CD45^+$ cells.

	WT	APP/PS1
Total counts	652.22	1255.29
% Total Cell Proportion	17.69	22.67
% Macrophages	27.54	40.76
% Effector CD8+ T cells	15.13	10.78
% Memory CD8+ T cells	1.32	1.79
% Memory CD4+ T cells	2.17	0.67
% Naive B cells	0.55	3.85
% Neutrophils	1.45	4.88
% ISG expressing immune cells	13.04	6.70
% Natural killer cells	1.74	4.13
% Erythroid like and erythroid precursor cells	3.85	3.88
% γδ T cells	5.88	2.15
% Progenitor cells	9.62	24.56
% Pre B cells	0.00	0.00
% Myeloid Dendritic cells	45.83	48.72
% Basophils	0.00	8.33

Supplementary Table S2. *Cxcl16* expression counts and percentages in scRNA-seq brain-derived CD45^{+} cells. The table lists the total counts, the total cells proportions and the percentages of expression of *Cxcl16* in the different cell clusters automatically identified in Van Hove *et al*., 2019 dataset, between WT and APP/PS1 brain-derived CD45^{+} cells.

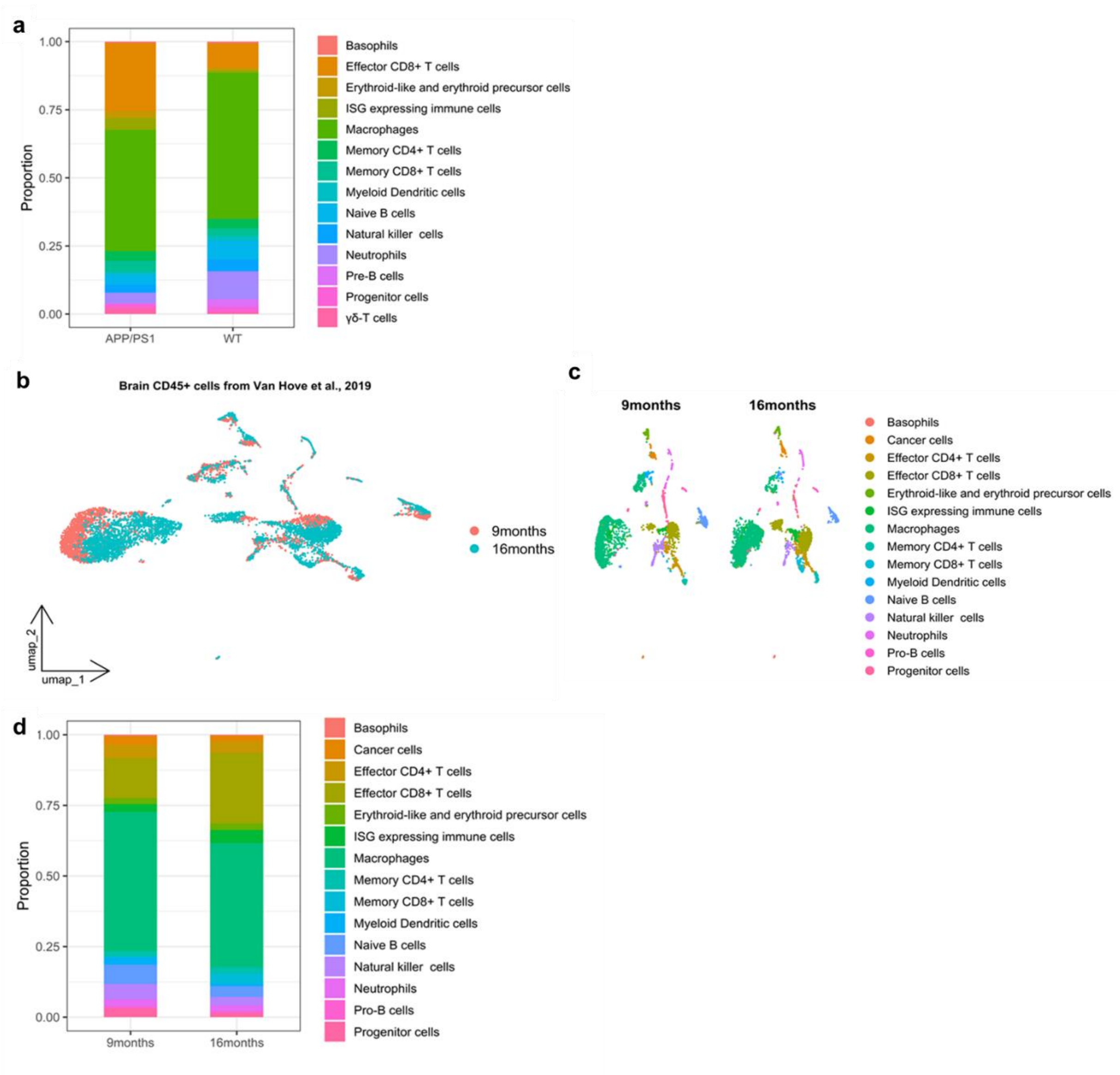

Supplementary Figure S1. scRNA-seq analysis of CD45$^+$ cells in APP/PS1 and WT mouse brain. a, **d**) Average cell proportions of each cluster in APP/PS1 and WT mice at 16 months of age (**a**) and in 9 and 16 months old APP/PS1 animals (**d**), in Van Hove *et al*., 2019. **b**) UMAP showing the clustering of CD45$^+$ brain-derived cells from APP/PS1 animals at 9 and 16 months old animals in Van Hove *et al*., 2019. **c**) UMAP showing the color-coded clustering of the cells, subdivided in in 9 (left plot) and 16 months old (right plot) APP/PS1 animals.

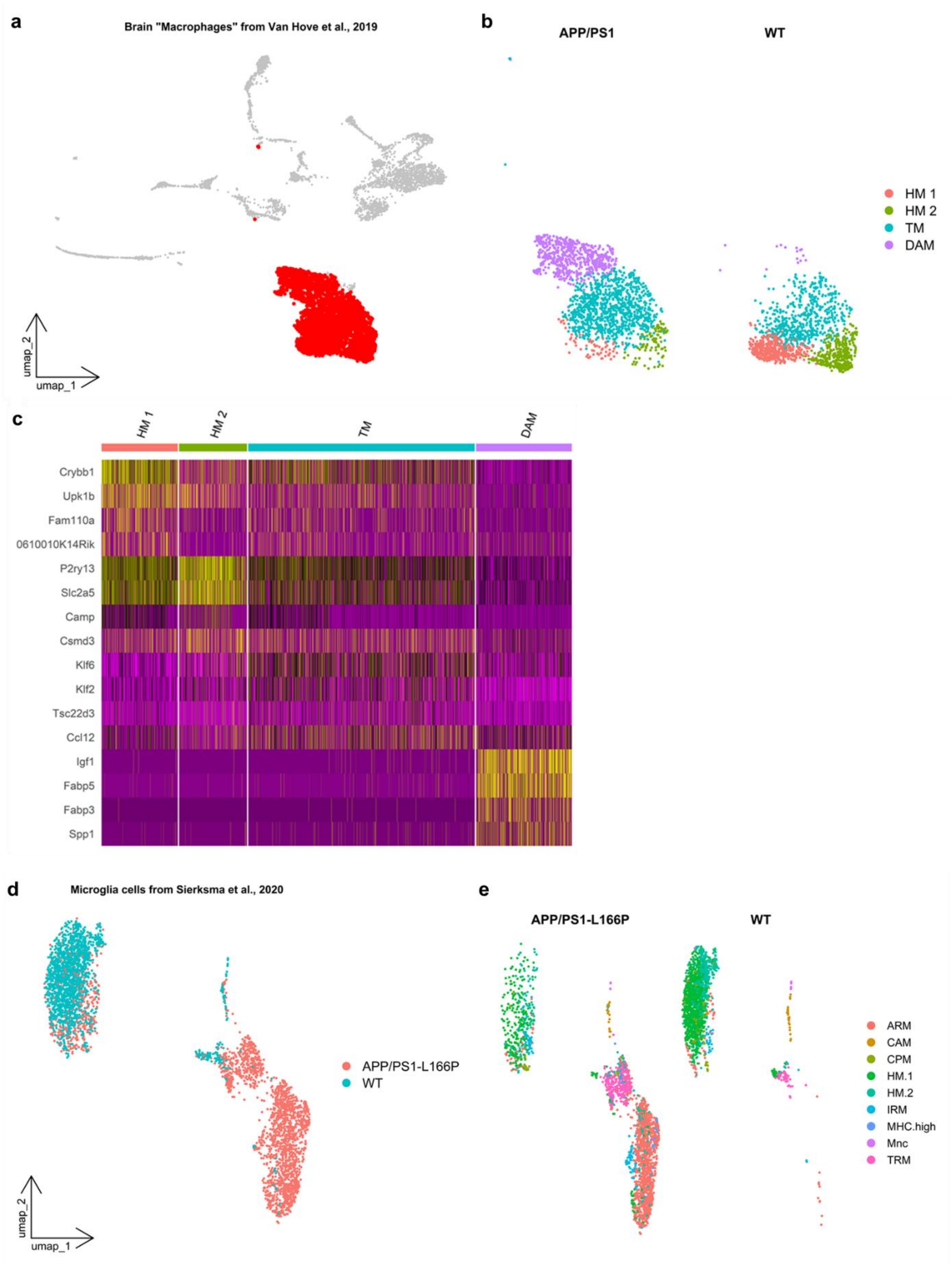

Supplementary Figure S2. Brain "Macrophages" sub-clustering of scRNA-seq from brain-derived CD45$^+$ cells in APP/PS1 mice and CD45$^+$/CD11b$^+$ cells in the hippocampus of APP/PS1-L166P. a) UMAP showing the "Macrophages" cluster in red of CD45$^+$ brain-derived cells from Van Hove *et al*., 2019. **b**) UMAP showing the color-coded sub-clustering of

“Macrophages” cells, subdivided in APP/PS1 (left plot) and WT (right plot). The identified sub-clusters are: “Homeostatic Microglia 1” (HM 1), “Homeostatic Microglia 2” (HM 2), “Transitioning Microglia” (TM) and “Disease-associated Microglia” (DAM). **c**) Heat-map showing the top four markers identifying the four sub-clusters. The color scale goes from yellow (highest expression) to purple (lowest expression). **d**) UMAP showing clustering of microglia cells from 11 months APP/PS1-L166P and WT animals in Sierksma *et al.*, 2020. **e**) UMAP showing the color-coded clustering of the cells: “Activated Response Microglia” (ARM), “CNS-Associated Macrophages” (CAM), “Cycling and Proliferating Microglia” (CPM), “Homeostatic Microglia Cluster 1” (HM.1), “Homeostatic Microglia cluster 2” (HM.2), “Interferon-Response Microglia” (IRM), “High Major Histocompatibility Complex-expressing Microglia” (MHC.high), “Monocytes” (Mnc), “Transitioning Microglia” (TRM), subdivided in APP/PS1-L166P (left plot) and WT (right plot).

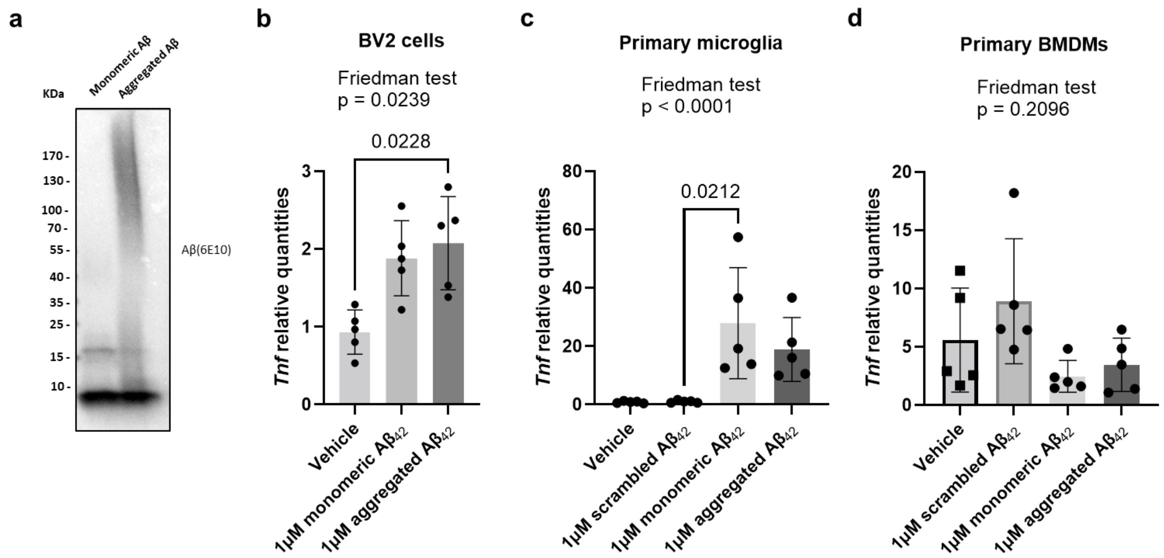

Supplementary Figure S3. Establishment of AD-like conditions in BV-2 cells. a) Representative Western Blot images of 2 µg of $A\beta_{42}$ ("monomeric" at 4-5 kDa) and 2 µg $A\beta_{42}$ after overnight incubation at 37 °C ("aggregated", characterized by the presence of higher molecular weight bands). **b-d)** qPCR analysis for *Tnf* mRNA expression level ("relative quantities" to medium-treated cells) in BV-2 cells treated with vehicle, 1 µM monomeric and 1 µM aggregated $A\beta_{42}$ (**b**), in primary microglia (**c**) and BMDMs (**d**) treated with vehicle, 1 µM scramble $A\beta_{42}$, 1 µM monomeric and aggregated $A\beta_{42}$ (n = 5 repeated experiments). Statistical analysis was performed with the Friedman test with Dunn's multiple comparison test (exact *p* values were calculated, and *p* values < 0.05 were considered as statistical significant and displayed in the plots).

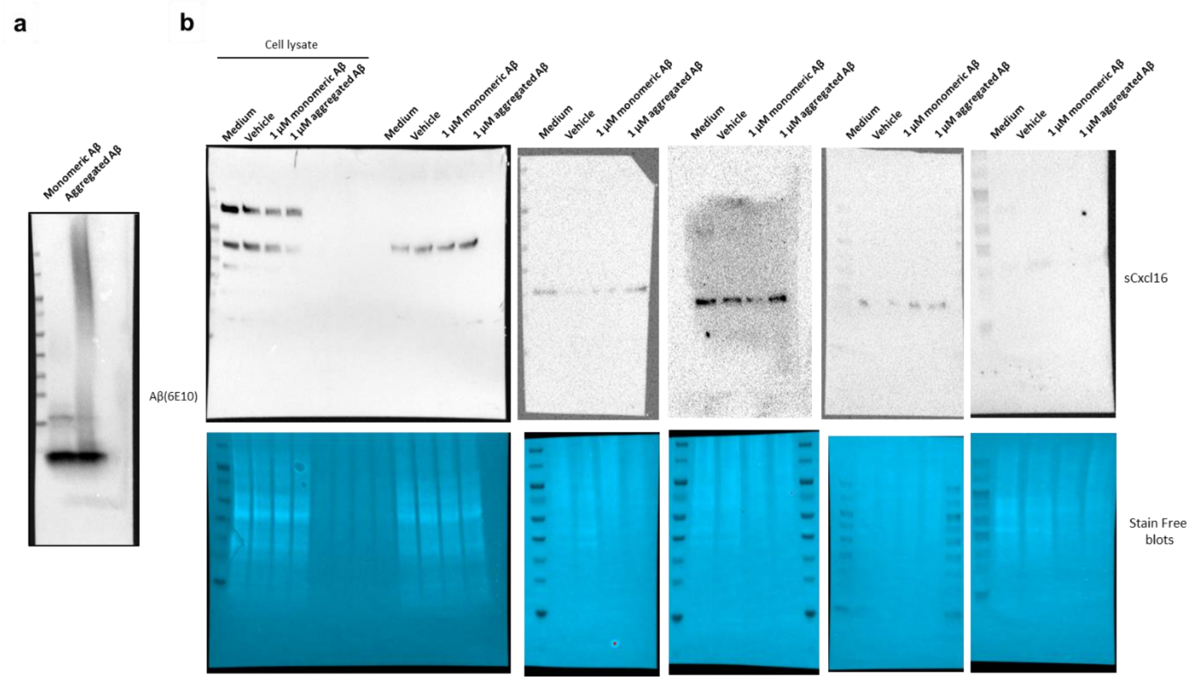

Supplementary Figure S4. Original Western Blot images. a, b) Full uncropped Western Blot images of monomeric and aggregated Aβ_{42} (**a**) and of intracellular Cxcl16 from cell lysate and secreted Cxcl16 (sCxcl16) in supernatants from BV-2 cells treated with medium alone, vehicle, 1 μM monomeric and 1 μM aggregated Aβ_{42} for 24 h. Full uncropped Stain-free Blot signals of medium sample for protein loading control of the Western Blot for sCxcl16 (**b**).

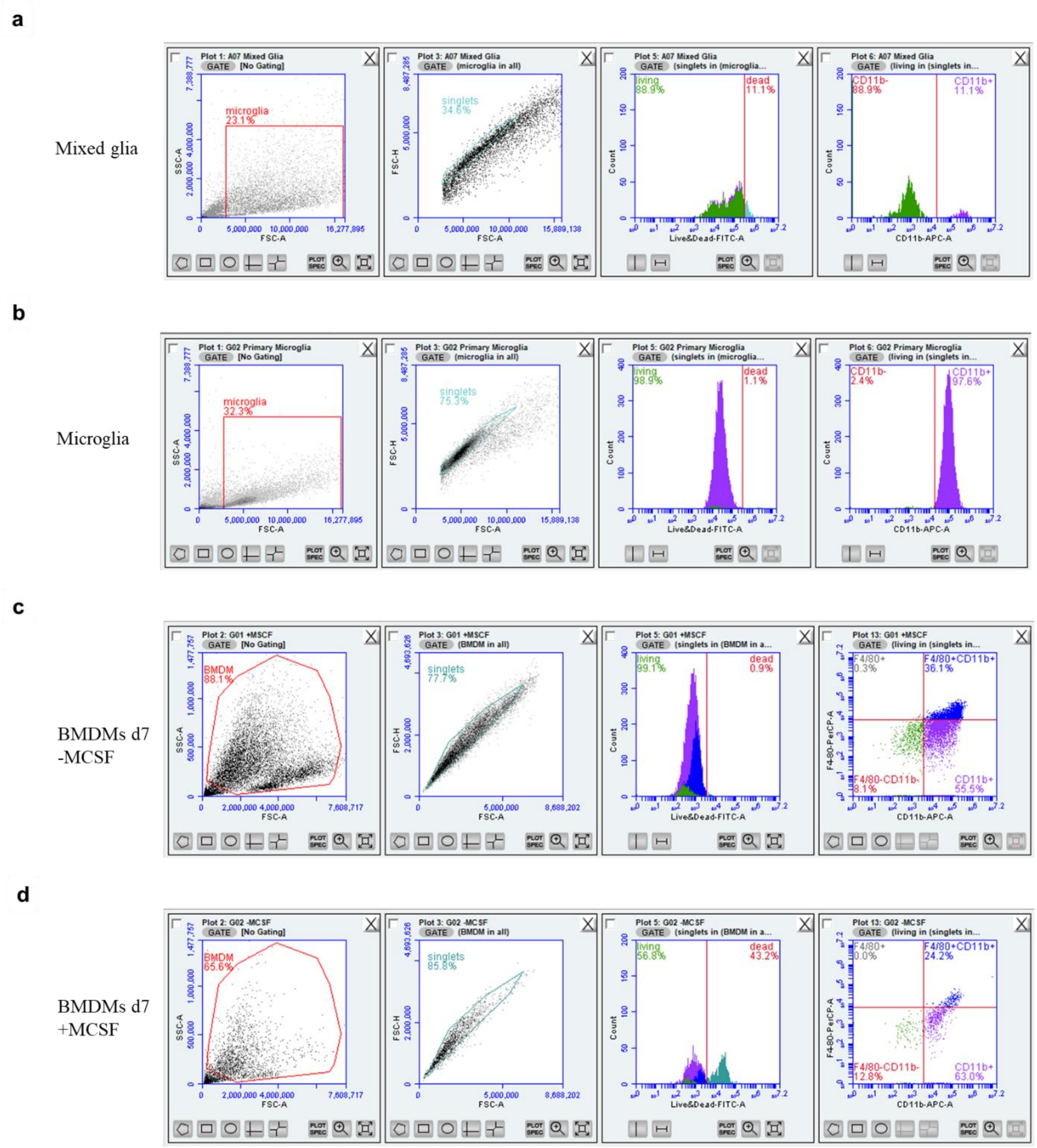

Supplementary Figure S5. Flow cytometry analysis of primary murine microglia and bone marrow-derived macrophages (BMDMs). a, **b**) Gating strategy for live mixed glial cells (**a**) and microglia (**b**), identified as $CD11b^{+}$ and negative for the Live/Dead dye. **c**, **d**) Gating strategy for live BMDMs after 7 days in culture without (**c**) or with 10 ng/mL macrophage colony-stimulating factor (M-CSF) (**d**). Over 90 % of BMDMs were $CD11b^{+}$, and within this population a subset co-expressed F4/80, indicating a double–positive $CD11b^{+}F4/80^{+}$ phenotype**.**

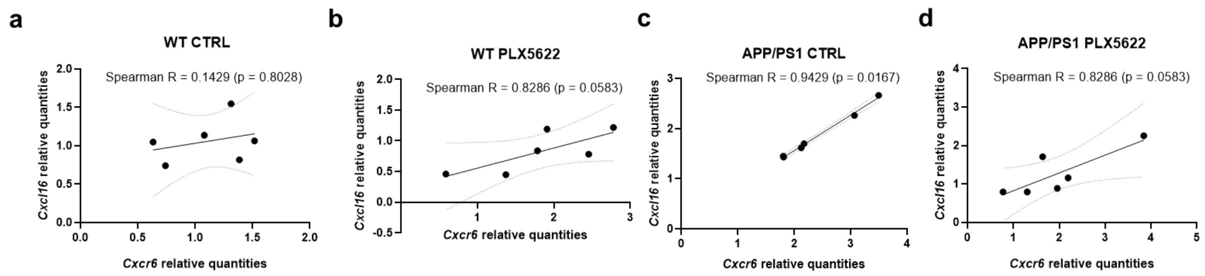

Supplementary Figure S6. *Cxcl16* and *Cxcr6* gene expression analysis in the hippocampus of control- and PLX5622-treated animals. a-d) Correlation analysis between *Cxcl16* and *Cxcr6* relative quantities in WT CTRL (**a**), WT PLX5622 (**b**), APP/PS1 CTRL (**c**) and APP/PS1 PLX5622 (**d**) animals. Spearman R correlation analysis was performed, and the exact *p* value was calculated.

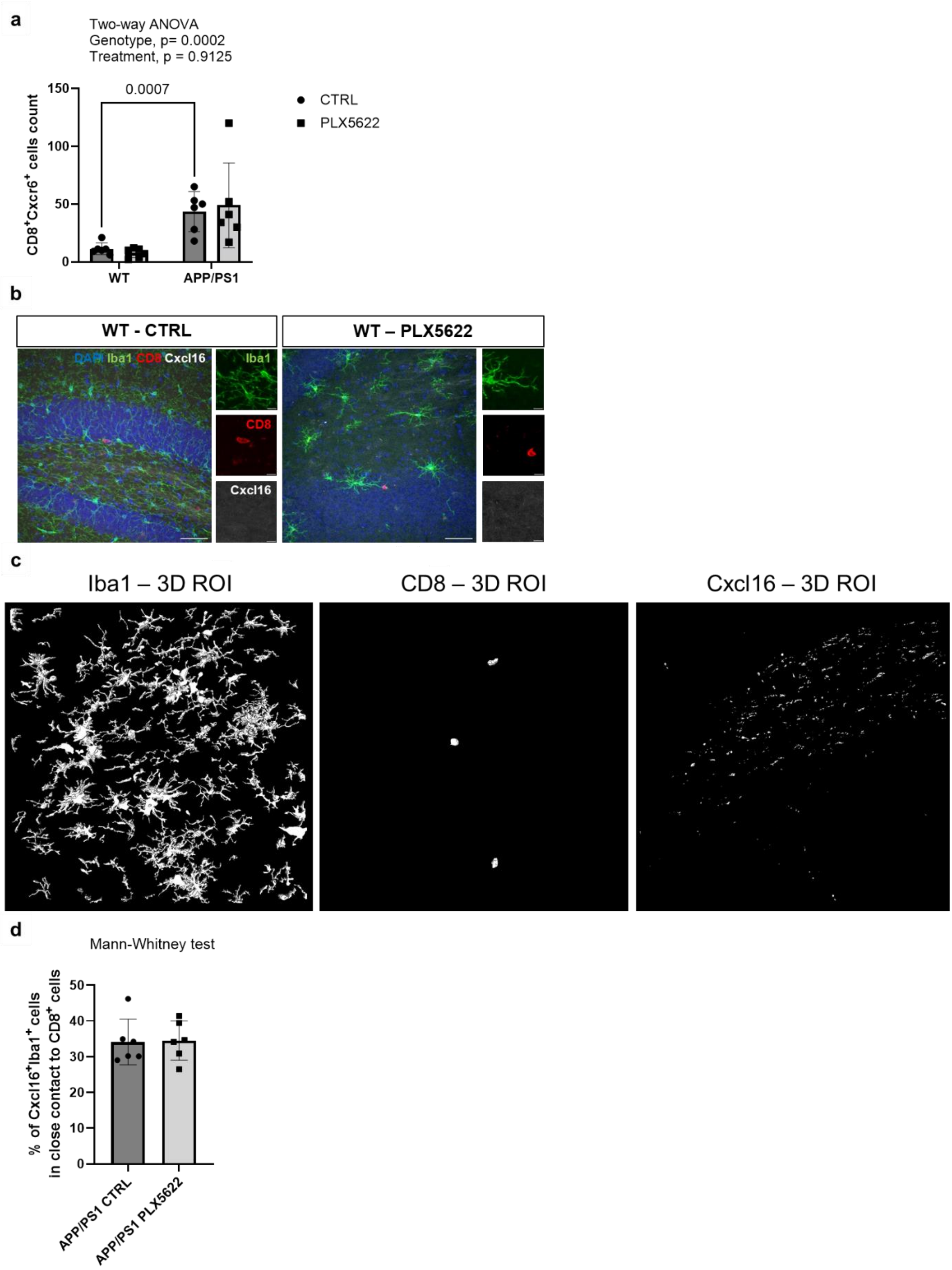

Supplementary Figure S7. Additional immunohistochemistry analysis in the hippocampus of control- and PLX5622-treated animals. a) Quantitative analysis of the total CD8$^+$ cells number expressing Cxcr6) in the hippocampal region of control and PLX5622-treated WT and APP/PS1 mice (n = 6 each group). Statistical analysis was performed with the

Two-way ANOVA and the exact *p* values were calculated. **b**) Representative IHC images of granule cell and polymorph layers of the dentate gyrus in hippocampal region of PLX5622-treated and control WT animals. Cxcl16 signal is in white, $CD8^+$ cells are in red, DAPI (blue) stains the nucleus, and Iba1 (green) is used to visualize microglia cells. Scale bar full picture: 50 µm, scale bar zoom: 10 µm. **c**) Representative of three-dimensional region of interests (3D ROIs) reconstruction for Iba1, Cxcl16 and CD8 using 3D manager in ImageJ. **d**) Quantitative analysis of the percentages of $Iba1^+$ cells expressing Cxcl16 with less 100 µm distance to $CD8^+$ cells in the hippocampal region of control and PLX5622-treated APP/PS1 mice (n = 6 each group). Statistical analysis was performed with the Mann-Whitney test.

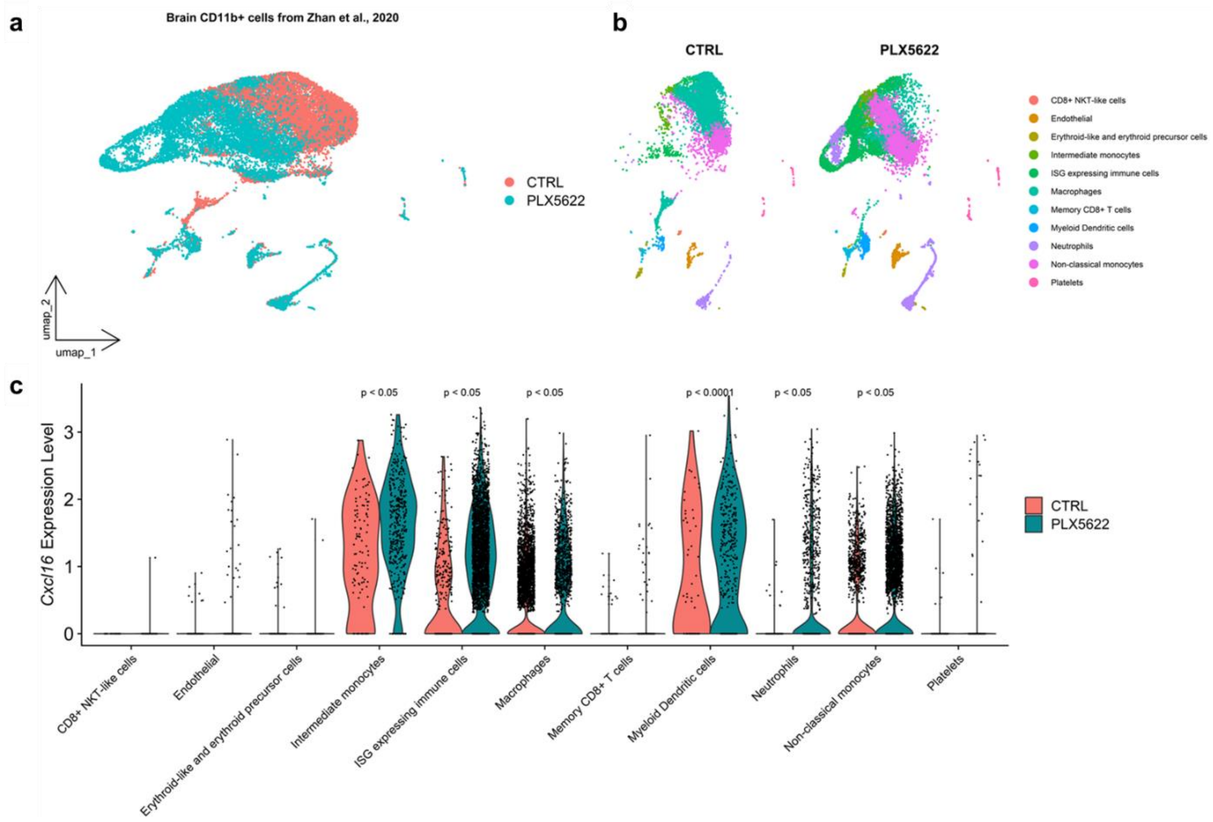

Supplementary Figure S8. PLX5622 effect on brain Cxcl16 expression. a) UMAP showing the color-coded clustering of CD11b^{+} brain-derived cells from control and PLX5622-treated WT animals in Zhan *et al*., 2020. **b**) UMAP showing the color-coded clustering of the cells, subdivided in PLX5622 (left plot) and CTRL (right plot). **c**) Violin plot showing the expression of *Cxcl16*, between CTRL and PLX5622 animals, in the different cell clusters. The dots indicate the cells, expressing the transcript, within each cluster. Statistical analysis was performed with Wilcoxon test. $p < 0.05$ was considered statistically significant.

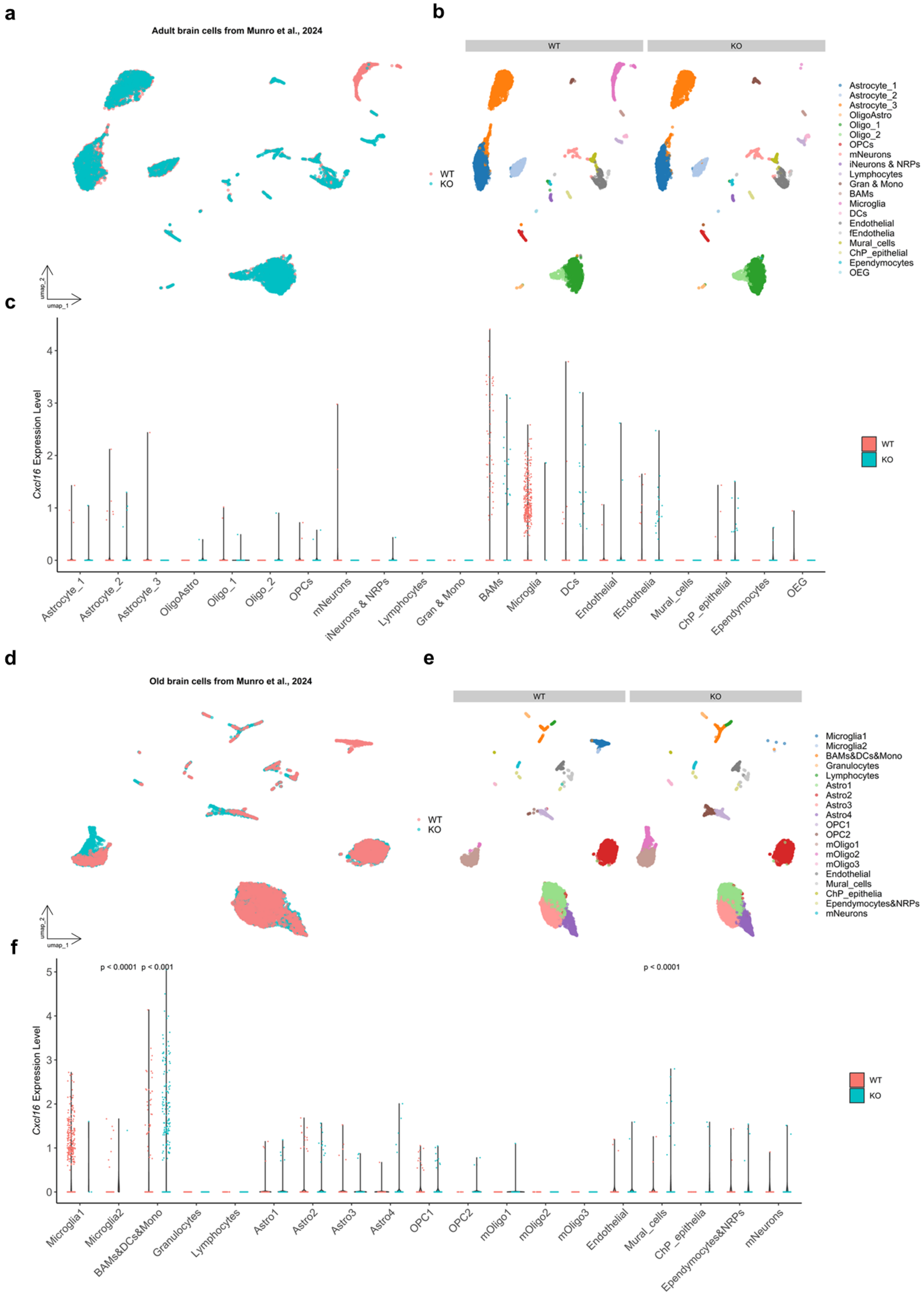

a
Adult brain cells from Munro et al., 2024
WT
KO
umap_2
umap_1
b
WT
KO
Astrocyte_1
Astrocyte_2
Astrocyte_3
OligoAstro
Oligo_1
Oligo_2
OPCs
mNeurons
iNeurons & NRPs
Lymphocytes
Gran & Mono
BAMs
Microglia
DCs
Endothelial
fEndothelia
Mural_cells
ChP_epithelial
Ependymocytes
OEG
c
Cxcl16 Expression Level
WT
KO
d
Old brain cells from Munro et al., 2024
WT
KO
umap_2
umap_1
e
WT
KO
Microglia1
Microglia2
BAMs&DCs&Mono
Granulocytes
Lymphocytes
Astro1
Astro2
Astro3
Astro4
OPC1
OPC2
mOligo1
mOligo2
mOligo3
Endothelial
Mural_cells
ChP_epithelia
Ependymocytes&NRPs
mNeurons
f
p < 0.0001
p < 0.001
p < 0.0001
Cxcl16 Expression Level
WT
KO

Supplementary Figure S9. Cxcl16 expression in brain cells after genetic targeting of Csf1r. a, d) UMAP showing the color-coded clustering of adult (11-12-month-old, **a**) and old (16-18-month-old, **d**) brain-derived cells from Csf1r$^{WT/WT}$ (WT) and Csf1r$^{\Delta FIRE/\Delta FIRE}$ (KO) animals in Munro *et al*., 20204. **b, e**) UMAP showing the color-coded clustering of the cells from adult (**b**) and old (**e**) animals, subdivided by genotype (WT, left plot and KO, right plot). "Oligodendrocytes and Astrocytes" (OligoAstro), "mature Oligodendrocytes" (Oligo_1, Oligo_2, or mOligo1-3), "Oligodendrocyte Precursor Cells" (OPCs or OPC1-2), "mature Neurons" (mNeurons), "immature Neurons and Neuronal Restricted Precursor cells" (iNeurons & NRPs), "Granulocytes and Monocytes" (Gran & Mono), "Border-Associated Microglia" (BAMs), "Dendritic Cells" (DCs), "Fibrotic Endothelial cells" (fEndothelia), "Choroid Plexus Epithelial cells" (ChP_Epithelial), "Border-Associated Microglia, Dendritic Cells and Monocytes" (BAMs&DCs&Mono), "Astrocytes" (Astro1-4), "Ependymocytes and Neuronal Restricted Precursor cells" (Ependymocytes&NRPs). **c, f**) Violin plot showing the expression of *Cxcl16*, between WT and KO animals, in the different cell clusters of adult (**c**) and old (**f**) animals. The dots indicate the cells, expressing the transcript, within each cluster. Statistical analysis was performed with Wilcoxon test. $p < 0.05$ was considered statistically significant and displayed in the plot.
